# Studies on the Virome of the Entomopathogenic Fungus *Beauveria bassiana* Reveal Novel dsRNA Elements and Mild Hypervirulence

**DOI:** 10.1371/journal.ppat.1006183

**Published:** 2017-01-23

**Authors:** Ioly Kotta-Loizou, Robert H. A. Coutts

**Affiliations:** 1 Department of Life Sciences, Faculty of Natural Sciences, Imperial College London, London, United Kingdom; 2 Geography, Environment and Agriculture Division, Department of Biological and Environmental Sciences, School of Life and Medical Sciences, University of Hertfordshire, Hatfield, United Kingdom; Mississippi State University, UNITED STATES

## Abstract

The entomopathogenic fungus *Beauveria bassiana* has a wide host range and is used as a biocontrol agent against arthropod pests. Mycoviruses have been described in phytopathogenic fungi while in entomopathogenic fungi their presence has been reported only rarely. Here we show that 21.3% of a collection of *B*. *bassiana* isolates sourced from worldwide locations, harbor dsRNA elements. Molecular characterization of these elements revealed the prevalence of mycoviruses belonging to the *Partitiviridae* and *Totiviridae* families, the smallest reported virus to date, belonging to the family *Narnaviridae*, and viruses unassigned to a family or genus. Of particular importance is the discovery of members of a newly proposed family Polymycoviridae in *B*. *bassiana*. Polymycoviruses, previously designated as tetramycoviruses, consist of four non-conventionally encapsidated capped dsRNAs. The presence of additional non-homologous genomic segments in *B*. *bassiana* polymycoviruses and other fungi illustrates the unprecedented dynamic nature of the viral genome. Finally, a comparison of virus-free and virus-infected isogenic lines derived from an exemplar *B*. *bassiana* isolate revealed a mild hypervirulent effect of mycoviruses on the growth of their host isolate and on its pathogenicity against the greater wax moth *Galleria mellonella*, highlighting for the first time the potential of mycoviruses as enhancers of biocontrol agents.

## Author Summary

Virus diversity is constantly increasing and the findings often challenge our perceptions of the virosphere, enhance our understanding of the global ecology and evolution of viruses and virus-like elements and offer a range of new tools. Here we describe a fungal viral family, provisionally designated as Polymycoviridae, with a variable number of genomic segments. Polymycoviruses contain at least four and up to seven genomic segments; the three smallest ones are individual and non-homologous among different members of the family, demonstrating an unprecedented dynamic genomic organisation in terms of both segment number and sequence. Additionally we report the smallest known virus to date, belonging to the family *Naranaviridae* which comprise the simplest unencapsidated RNA viruses. Finally, we illustrate for the first time the potential of mycoviruses as enhancers of the biological control agent *Beauveria bassiana*, an entomopathogenic fungus utilised as an environmentally friendly alternative to chemical insecticides.

## Introduction

*Beauveria bassiana* (Balsamo) Vuillemin is an entomopathogenic ascomycete belonging to the family *Clavicipitaceae*, order *Hypocreales*. It has a wide host range of approximately 750 arthropod species and a widespread geographical distribution. *B*. *bassiana* isolates have also been recovered from soil and several plant species as endophytes [1]. Specific *B*. *bassiana* strains, such as GHA and ATCC 74040, are available commercially as biocontrol agents against a variety of arthropod pests [2]. Although mycoinsecticides constitute an environmentally friendly, relatively cost-effective alternative to chemical insecticides, currently they are not widely used largely due to a failure of identifying strains consistently active at low doses that eliminate pests rapidly [3].

Mycoviruses have been described in a wide range of fungi and are classified into eleven major families, six accommodating single-stranded (ss) and five accommodating double-stranded (ds) RNA genomes. The former group includes the families *Narnaviridae* and *Hypoviridae*, while the latter includes the families *Totiviridae* and *Partitiviridae*. Recently an increasing number of novel mycoviruses have been reported including a negative-strand RNA mycovirus [4], a geminivirus-related DNA mycovirus [5], and a novel family Tetramycoviridae was proposed to accommodate a non-conventionally encapsidated mycovirus with four dsRNA segments as its genome that is infectious as dsRNA [6]. In *B*. *bassiana* the presence of dsRNA elements, virus-like particles and associated hypovirulence have been reported [7,8]; however only two viruses belonging to the genus *Victorivirus*, family *Totiviridae* have been sequenced [9,10].

In the present study all mycoviruses and other dsRNA elements found in a large collection of *B*. *bassiana* isolates were characterised. These findings include a description of the smallest virus reported up to date, new members of the established families *Partitiviridae* and *Totiviridae* and the proposed novel family Tetramycoviridae now renamed Polymycoviridae which appear to have an unprecedented dynamic nature in terms of genomic element number and sequence. Additionally, the potential of mycoviruses as enhancers of the biocontrol agent *B*. *bassiana* is demonstrated.

## Results and Discussion

### Incidence of dsRNA elements in *Beauveria bassiana*

In order to assess the presence of dsRNA elements in *B*. *bassiana*, we screened a well-characterized panel of isolates sourced from worldwide locations [11]. This population study revealed that 16/75 (21.3%) *B*. *bassiana* isolates harbor unique nucleic acid elements following electrophoretic separation on agarose gels (S1A Fig; S1 Table). Resistance to DNase 1 and to RNase A treatment in high salt, but sensitivity to RNase III and RNase A in low salt confirmed the dsRNA nature of these elements. Eleven of the *B*. *bassiana* isolates harboring dsRNA elements were recovered directly from arthropods, while the rest were collected from soil and their preferred host is unknown (S1 Table). There is no correlation between the presence of dsRNA and the fungal isolates’ arthropod host, geographical origin, microenvironment or evolutionary relationship. Subsequently, the isolates were grouped according to their dsRNA banding patterns and exemplar isolates were investigated further.

### Partitiviruses and victoriviruses

The electrophoretic patterns and sizes of the dsRNAs described in S1A Fig suggest that seven *Beauveria bassiana* isolates, IMI 331273, IMI 386705, IMI 391044, IMI 391704, IMI 392612, EABb 00/88Su and EABb 00/23Su likely harbor partitiviruses. Two isolates, IMI 331273 and IMI 392612, were selected for further analysis, as representatives of two distinct groups exhibiting slightly different dsRNA profiles (S2A Fig). Virus particles were isolated from both isolates and visualised by electron microscopy as isometric particles 50 nm in diameter (S2B Fig).

The genome of the virus derived from isolate IMI 331273 consists of two dsRNA segments, 1771 bp and 1601 bp in size (S2C Fig). The 1771 bp segment contains a single open reading frame (ORF) potentially encoding a protein of 539 amino acids (aa; 63 kDa) flanked by 5’- and 3’-untranslated regions (UTRs). The 1601 bp segment potentially encodes a protein of 440 aa (47.1 kDa). The 5’-UTRs of the two segments are 68 and 101 bp in size while the 3’-UTRs are 83 and 177 bp in size respectively. The 5’-terminal sequences of the two dsRNAs are identical (CGCAAA) and the 3’-terminal sequences are very similar (AGATCA for the 1771 bp segment and AACTCA for the 1601 bp segment). The genome organisation of the viruses derived from isolates IMI 392612 and 331273 are similar (S2C Fig) and its two dsRNA segments, 1801 bp and 1548 bp, potentially encode proteins of 539 aa (62.7 kDa) and 432 aa (46.8 kDa), respectively. The 5’-UTRs of the two segments are 64 and 122 bp in size while the 3’-UTRs are 117 and 127 bp in size respectively. The 5’-terminal sequences of the two dsRNAs are identical (CGCAAAA) and very similar to those of the dsRNAs in isolate 331273, while the 3’-terminal sequences are identical (AAAATCCA).

BLAST searches of the viral proteins revealed significant similarities to established and tentative members of the family *Partitiviridae*. Therefore, the viruses derived from isolates IMI 331273 and IMI 392612 were named Beauveria bassiana partitivirus (BbPV)-1 and -2, respectively. Analysis of the BbPV-1 and BbPV-2 RdRP aa sequences revealed typical motifs (S2D Fig) and a phylogenetic tree of established partitiviruses showed that both BbPV-1 and -2 cluster with members of the genus *Gammapartitivirus* which exclusively infect fungi (S2E Fig), in contrast to the genera *Alphapartitivirus* and *Betapartitivirus*, which include viruses that infect plants and fungi, the genus *Deltapartitivirus*, which exclusively infect plants, and the genus *Cryspovirus*, which infect protozoa [12]. Since BbPV-1 and BbPV-2 are not sister taxa, partitiviruses appear to have been introduced into *B*. *bassiana* more than once. BbPV-2 was found in Europe, Asia and South America and has a wider geographical distribution than BbPV-1, which was restricted to South America and the Canary Islands (S1 Table; S1B Fig).

Comparison of the BbPV-1 and -2 RdRP and CP sequences revealed 70% identity at both nucleotide and protein levels for the former and 67% and 43% identity for the latter. DIG-labelled probes that do not cross-react were generated from the RdRP and the CP gene sequences of both partitiviruses and northern blot hybridizations were performed to confirm partitivirus presence in the remaining five *B*. *bassiana* isolates. As expected, based on electrophoretic profiles, two distinct partitivirus groups were detected; isolates IMI 386705 and EABb 00/23Su harbor partitiviruses very similar to BbPV-1, while isolates IMI 391044, IMI 391704 and EABb 00/88Su harbor partitiviruses very similar to BbPV-2 (S2F Fig).

Interestingly, isolates EABb 00/23Su and EABb 00/88Su apparently harbor mixed infections; both containing a partitivirus, and a larger dsRNA *ca*. 6 kbp in size, possibly a member of the genus *Victorivirus* in the family *Totiviridae*, as reported previously [9,10]. Indeed, partial amplification and sequencing of the RdRP gene (*ca*. 436 bp) confirmed the presence of victoriviruses in EABb 00/23Su, and EABb 00/88Su and in EABb 01/12Su and EABb 01/103Su (S3B Fig). All these victoriviruses were similar, but not identical, to Beauveria bassiana victorivirus (BbVV)-1 [9]. The nucleotide sequences of the amplicons derived from isolates EABb 01/103Su, EABb 01/12Su and EABb 00/88Su, which originate from the south of the Iberian Peninsula (S1 Table; S1B Fig), are identical and share 84% identity at the nucleotide sequence level, as well as 98% identity and 100% similarity at the aa sequence level with BbVV-1. Similarly, BbVV-1 and the amplicon derived from isolate EABb 00/23Su, found in Tenerife in the Canary Islands, have 84% identity at the nucleotide sequence level and 97% identity and 98% similarity at the aa sequence level.

### Polymycoviruses

**Fig 1:**
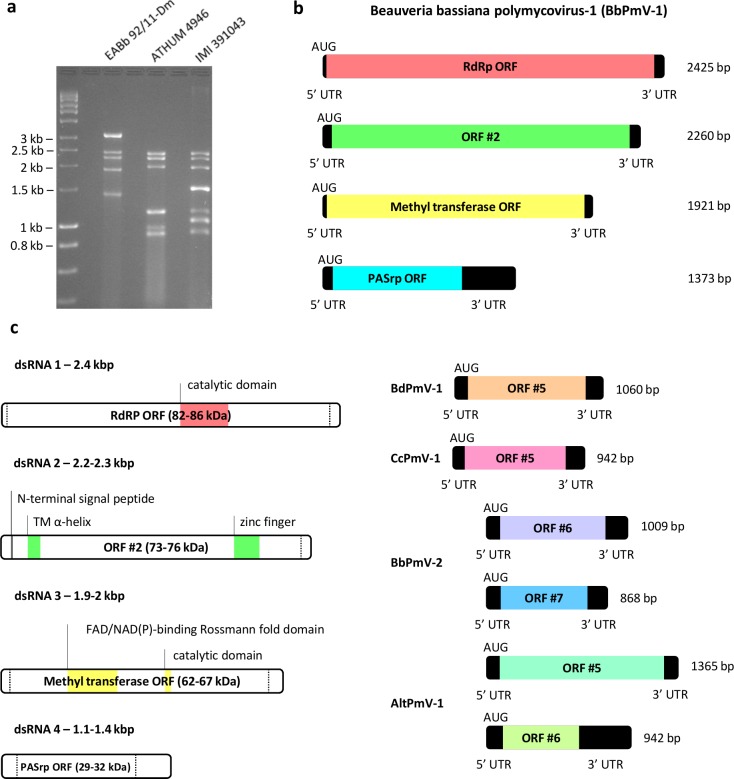
Polymycoviruses in *Beauveria bassiana*. **(a)** 1.5% (^w^/_v_) agarose gel electrophoresis of dsRNA extracted from *B*. *bassiana* isolates EABb 92/11-Dm, IMI 391043 and ATHUM 4946 harboring BbPmV-1, BbPmV-2 and BbPmV-3 respectively. Lane 1 contains the DNA marker Hyperladder I (Bioline), the sizes of which are shown to the left of the gel. **(b)** Schematic representation of the genomic organization of BbPmV-1. The BbPmV-1 genome consist of four dsRNAs, each containing one ORF (grey boxes) flanked by 5’- and 3’-UTRs (black boxes). **(c)** Schematic representation of the genomic organization of the proposed family Polymycoviridae illustrating known or predicted features of the proteins encoded by the four major dsRNAs. Additional sequenced elements, unique for selected polymycoviruses, each containing one ORF (grey boxes) flanked by 5’- and 3’-UTRs (black boxes) are shown and include BdPmV-1 dsRNA 5, CcPmV-1 dsRNA 5, BbPmV-2 dsRNAs 6 and 7, and AltPmV-1 dsRNAs 5 and 6.

*B*. *bassiana* isolates EABb 92/11-Dm, ATHUM 4946, IMI 391043, IMI 391362, SP R 184 and SP U 259 all harbor a large number of dsRNA elements, 0.8–3.1 kbp in size, three of which are shown (Fig 1). The sequence of the largest dsRNA in EABb 92/11-Dm, designated Beauveria bassiana non-segmented virus (BbNV)-1 has been described previously in this isolate [13] and in *B*. *bassiana* isolate A24 [14]. BbNV-1 has two overlapping ORFs [15] that encode a protein of unknown function and an RdRP, respectively, and was provisionally designated as a member of a new proposed viral family Unirnaviridae [13].

The remaining segments 2425, 2260, 1921 and 1373 bp in size each respectively contain an ORF on the plus-strand potentially encoding proteins 755 aa (86 kDa), 704 aa (75 kDa), 610 aa (67 kDa) and 306 aa (32 kDa) in size, flanked by 5’- and 3’-UTRs. The 5’-UTRs of the four segments are respectively 26, 71, 31 and 72 bp long while the 3’-UTRs are 71, 74, 57 and 380 bp long. The 5’- and 3’-terminal sequences of all four dsRNAs are very similar (S4F Fig). Partial analysis of the dsRNA elements from ATHUM 4946 and IMI 391043 revealed that these isolates also harbor segments homologous to those found in EABb 92/11-Dm. As expected based on their electrophoretic profiles and as demonstrated by northern blotting, isolates IMI 391362, SP R 184 and SP U 259 harbor dsRNA elements very similar to those found in IMI 391043.

BLAST searches of the viral proteins revealed that the dsRNA segments constitute the genome of viruses belonging to a newly proposed family, the Tetramycoviridae, the prototype member of which is Aspergillus fumigatus tetramycovirus-1 (AfuTmV-1) [6]. However, taking into account that two of the viruses described here have six and seven segments, respectively, together with the existence in the databases of mycoviruses from *Cladosporium cladosporioides* and *Botryosphearia dothidea* [16] each with five segments and belonging to the same family (S4A and S4B Fig), we now propose to rename this family as Polymycoviridae (*poly* = ‘many’ in Greek, in contrast to *tetra* = ‘four’). Therefore, the viruses derived from isolates EABb 92/11-Dm, IMI 391043 and ATHUM 4946 were designated as Beauveria bassiana polymycovirus (BbPmV)-1, -2 and -3 respectively. Following a search of the public databases, all complete or partial sequences of putative polymycoviruses were collected (S3 Table) and analysed together. A general characteristic of the polymycovirus genome is the high GC content, ranging from 57% for *C*. *cladosporioides* (Cc) PmV-1 to 63% for AfuPmV-1. The RdRPs, encoded by the largest dsRNA1 segments of all the proposed members of the Polymycoviridae family, contain three partially conserved motifs (S5A Fig) found in the picorna-like RdRP family of positive-strand, RNA eukaryotic viruses (RdRP_1, PF00680). As described for AfuPmV-1 [6] and now all polymycoviruses the GDD motif and catalytic site of the RdRP has been replaced with a GDQN motif, normally characteristic of negative-strand ssRNA viruses of the order *Mononegavirales*. Phylogenetic analysis of the polymycovirus RdRP sequences confirmed close evolutionary proximity to members of the families *Caliciviridae* and *Astroviridae* (S6 Fig) which are non-enveloped, non-segmented, positive-sense ssRNA viruses that infect vertebrates, particularly mammals and birds. Interestingly, polymycoviruses originating from distantly related fungal hosts appear to be evolutionary close. For example, AfuPmV-1 from *A*. *fumigatus* (class *Eurotiomycetes*), BdPmV-1 from *B*. *dothidea* (class *Dothideomycetes*) and BbPmV-2 (class *Sordariomycetes*) group together, while the two *B*. *bassiana* polymycoviruses do not. This may indicate that host shifts are quite frequent for polymycoviruses.

The proteins encoded by polymycovirus dsRNA2 segments contain a cysteine-rich, zinc finger-like motif (S5B Fig) and, unlike the rest of the viral proteins have a remarkably conserved N-terminus (MADLT/ARL). The MEMSAT-SVM algorithm [17] predicts the presence of a N-terminal signal peptide (residues 1–12) in all proteins encoded by the dsRNA2 segments, while both MEMSAT-SVM and TMPred [18] indicated the presence of a conserved transmembrane alpha-helix at their N-terminal domains (between residues 40–70). Interestingly, all proteins encoded by the dsRNA2 segments are rich in arginine repeats (R-R, R-X-R, R-R-R), associated with endoplasmic reticulum (ER) retention signals normally found in transmembrane proteins. Many positive-sense ssRNA viruses, including caliciviruses [19] and astroviruses [20] replicate in association with ER membranes and encode transmembrane proteins with ER retention signals. Notably, the usual target peptide KDEL sequence is not always discernible in viral proteins experimentally proven to be associated with the ER. Although the function and biological role of these proteins still remain unknown, the above data suggest that they may be implicated in virus replication as scaffolds for the replication machinery and/or as dsRNA chaperones.

The proteins encoded by the dsRNA3 segments contain a conserved catalytic methyltransferase motif (S5C Fig) and a N-terminal FAD/NAD (P) binding Rossmann-fold domain (Pfam clan CL0063) characteristic of methyltransferases. Methyltransferases are involved in the modification of the 5’-terminus of RNAs to form a cap structure and the presence of such at the 5’-terminus of the positive-strand of BbPmV-1 dsRNA1 was confirmed by oligo-cap analysis (S5C Fig), as described previously [6,21]. Conversely, the positive-strand of BbNV-1 is apparently uncapped, indicating that the two viruses employ different translation initiation methods–for example it is feasible that the long BbNV-1 5’-UTR may function as an IRES-like element–and this probably facilitates the co-existence of two mycoviruses in the same fungal host.

The proteins encoded by the dsRNA4 segments are proline-alanine-serine (PAS) rich (S5D Fig) and in the case of AfuPmV-1 [6] and BdPmV-1 [16] have been shown to be associated with the viral genome which is non-conventionally encapsidated. As anticipated BbPmV-1 is also non-conventionally encapsidated following atomic force microscopy visualisation (S5F Fig). Interestingly, the lengths of some of the chain-like, linear nucleic acids correspond to those predicted from the size of the BbNV-1 genomic dsRNA. Closely related viruses members of the proposed family Unirnaviridae including BbNV-1 could not be detected as conventional particles by TEM [14] and do not encode a CP but do encode a proline/alanine/serine (PAS)-rich protein (S5E Fig), which most likely coats the viral dsRNA in a similar fashion to the polymycoviruses.

Of particular interest are the small dsRNA segments isolated from the fungi harboring CcPmV-1, BdPmV-1, BbPmV-2 and AltPmV-1; the latter found in *Alternaria* sp. FA0703. Further analysis revealed that their 5’- and 3’- termini are very similar to those of the larger segments from the same isolate (S4C–S4E and S4G Fig), indicating that in each case all dsRNA elements constitute the genome of one virus. All the small segments have the capacity to encode proteins; however there is no detectable homology between these polypeptides, suggesting that polymycoviruses have a previously unreported dynamic genomic organisation in terms of segment number and sequence. Highly basic, intrinsically disordered (ID) PASrps are encoded by CcPmV-1 dsRNA5 (pI 11.4; 100% ID), AltPmV-1 dsRNA5 (pI 9.5; 66% ID) and BbPmV-2 dsRNA7 (pI 11.2; 94% ID), while acidic, ordered proteins are encoded by AltPmV-1 dsRNA6 (pI 6.5; 21% ID) and BdPmV-1 dsRNA5 (pI 4.9; 13% ID). Finally, BbPmV-2 dsRNA6 encodes a basic, ordered protein (pI 8.8; 6.4% ID) (S5G Fig).

None of these polypeptides have significant homology with sequences deposited in the public databases. The protein encoded by CcPmV-1 dsRNA5 contains a viral transcriptional regulator domain (CDD PHA03307; residues 15–165; E-value = 2.3e^-03^) and appears to be distantly related to fungal transcription factors with helix-loop-helix domains (identity 36%; similarity 54%; E-value = 0.9). Additionally the AltPmV-1 dsRNA5 product belongs to the nucleotide kinase protein superfamily (CDD cl17190; residues 34–128; 9.03e^-04^). The function and the biological role of these polypeptides are unknown, but since they are not present in all polymycoviruses they are probably not essential for virus replication and maintenance. In some mycovirus families e. g. “chryso-like” viruses, at least two divergent virus clades with either 3 or up to 5 genome segments have been described [22]. Additionally megabirnaviruses can replicate successfully without the smaller genomic dsRNA2 segment but it is required for efficient replication, maintenance in culture, and hypovirulence induction [23]. Alternatively, it is feasible that the non-conventionally encapsidated nature of the viruses allows them to incorporate host genes into their genomes which encode proteins important for viral replication rather than being dependent on the host machinery.

### A small Narna-like virus

**Fig 2:**
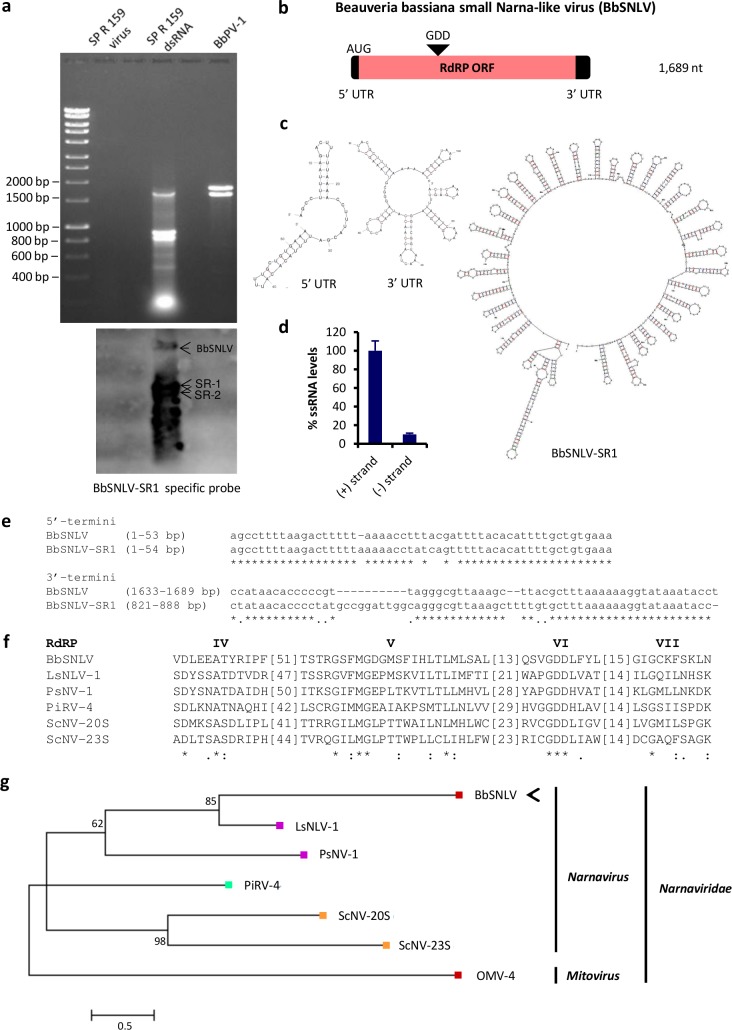
A small Narna-like virus in *Beauveria bassiana*. **(a)** 1% (^w^/_v_) agarose gel electrophoresis and northern blot hybridization of dsRNA extracted from *B*. *bassiana* isolate SP R 159, using virus purification (lane 2) and LiCl dsRNA extraction (lane 4). Lane 6 contains partitivirus BbPV-1, as a negative control. Hybridization was carried out using specific probes for BbSNLV-SR-1: BbSNLV and SR 1 and 2 are indicated by arrows. **(b)** Schematic representation of the genomic organization of BbSNLV. The BbSNLV genome consists of a single RNA containing one ORF (grey box) flanked by 5’- and 3’-UTRs (black boxes). **(c)** Predicted secondary structure of the BbSNLV 5’- and 3’-UTRs and the (+) strand of BbSNLV-SR-1 using mfold (folding temperature 25°C). **(d)** Relative RNA levels of the positive and negative strands BbSNLV-SRs within total fungal ssRNA extracts, as shown by RT-qPCR amplification. At least three independent repetitions were performed in triplicate and error bars represent standard deviation. **(e)** A comparative alignment of the 5’- and 3’-terminal sequences of BbSNLV and BbSNLV-SR-1. Asterisks signify identical nucleotides. **(f)** A comparison of the conserved motifs of the RdRP in BbSNLV and other narnaviruses (S2 Table). Numbers within the brackets indicate the number of aa not shown. Asterisks signify identical aa residues, colons signify highly conserved residues and single dots signify less conserved but related residues. **(g)** Maximum likelihood phylogenetic tree created based on the alignment of RdRP sequences of narnaviruses (S2 Table) using the LG+G+I+F substitution model. Branches with bootstrap support lower that 50% were collapsed. At the end of the branches, red, orange, green and purple indicate that the virus infects filamentous fungi, yeast, oomycetes and kinetoplastids respectively. BbSNLV is indicated by an arrow.

*Beauveria bassiana* isolate SP R 159 was found to harbor a range of dsRNA elements (Fig 2A). The three most abundant dsRNAs were cloned and sequenced; the largest one is 1,689 bp in size and has the potential to encode a 509 aa (58 kDa) polypeptide, flanked by a 53 bp 5’-UTR and a 106 bp 3’-UTR (Fig 2B). The polypeptide is distantly related to the RdRP of Leptomonas seymouri Narna-like virus 1 (LsNLV-1; PSI-BLAST; E-value 0.009, 25% identity, 41% similarity) and contains motifs characteristic of RdRPs, including the conserved GDD motif (Fig 2F). The virus was named Beauveria bassiana small Narna-like virus (BbSNLV), and a phylogenetic tree showed that it clusters with members of the genus *Narnavirus* of the family *Narnaviridae* (Fig 2G). To our knowledge BbSNLV constitutes the smallest known virus within the family of the simplest, non-segmented unencapsidated RNA viruses that range from 2.3 to 3.6 kb in size [24]. Additionally, BbSNLV is the first narnavirus isolated from a hyphomycete, the other 5 members of the genus being found in yeast (ScNV-20S and -23S) [25], an oomycete (PiRV-4) [26] and kinetoplastids (LsNLV-1 and PsNV-1) [27].

The other two prominent dsRNAs are 888 bp and 805 bp in size, identical in sequence (the foreshortened 5’ terminus of the 805 bp dsRNA notwithstanding), with no ORFs of significant length in either strand and no statistically significant similarity with other known sequences. Their termini are very similar to those of BbSNLV (Fig 2E), suggesting that they are satellite or defective RNAs dependent on BbSNLV for their replication. Both types of subviral RNAs are common in the *Narnaviridae* but restricted to the genus *Mitovirus* [24] and the large amounts found in BbSNLV infected isolates here suggest they might be defective interfering RNA by being derived from and reducing accumulation of the parent viral RNA (Fig 2A). Northern blot hybridization revealed that the remainder of the dsRNA species of the SP R 159 isolate are all very similar as they cross-hybridise (Fig 2A), while no DNA counterpart of these dsRNAs in the fungal genome was detected by Southern blotting or PCR amplification. Subsequently RT-qPCR amplification revealed a quantitative asymmetry between the positive and the negative strands (Fig 2D), as expected since narnaviruses are positive stranded ssRNA viruses, supporting the notion that the dsRNA detected and characterised constitutes the replicative form. Secondary structure analysis of the positive strands using the mfold server [28] predicted the formation of multiple stem-loops at both the 3’- and 5’-termini (Fig 2C) characteristic of all narnaviruses identified thus far [24].

### Mycovirus effects on fungal growth and virulence

In order to assess the effects of mycoviruses on the growth and virulence of the fungal host, isogenic lines of virus-free and virus-infected EABb 92/11-Dm isolate were generated. Initial attempts to cure EABb 92/11-Dm using protein synthesis inhibitors as described [8] and through single conidium isolation, failed. Subsequently a combinational approach was devised by growing the isolate on agar plates containing 150 mM cycloheximide in order to reduce the viral RNA levels and then performing single conidium isolation. The absence of both BbPmV-1 and BbNV-1 was confirmed by northern hybridization and RT-PCR amplification for BbPmV-1 (S7A and S7B Fig respectively) and RT-PCR amplification for BbNV-1 (S7C Fig) and three cured isolates were chosen, designated as EABb 92/11-DmC1, EABb 92/11-DmC2 and EABb 92/11-DmC3 for further studies. Interestingly, no colonies harboring just one of the viruses were recovered, suggesting that BbPmV-1 and BbNV-1 have similar sensitivity to cycloheximide. Similarly, no loss of any genomic segments from the multi-segmented BbPmV-1, BbPmV-2 or BbPmV-3 was noted after growing their respective fungal isolates on plates containing different concentrations of cycloheximide and performing single conidium isolation. However, no definitive conclusions can be drawn about the importance of each segment for the viral replication cycle based on this observation.

The growth rates of the isolates EABb 92/11-Dm, EABb 92/11-DmC1, EABb 92/11-DmC2 and EABb 92/11-DmC3 were compared both in solid and liquid Czapek-Dox complete medium. A small but statistically significant increase in radial growth and biomass production, respectively, was observed for the three virus-free strains in comparison to EABb 92/11-Dm (Student’s t test, P-value < 0.05; S7D and S7E Fig respectively). Moreover, the virulence of the virus-free and the virus-infected isolates was compared using the greater wax moth *Galleria mellonella*, as described before [29]. A similar small, but statistically significant decrease in the survival rates of the *G*. *mellonella* larvae infected with three virus-free strains in comparison to EABb 92/11-Dm was noted (Log-rank test, P-value < 0.05; Wilcoxon test, P-value < 0.05; S7F Fig).

**Fig 3:**
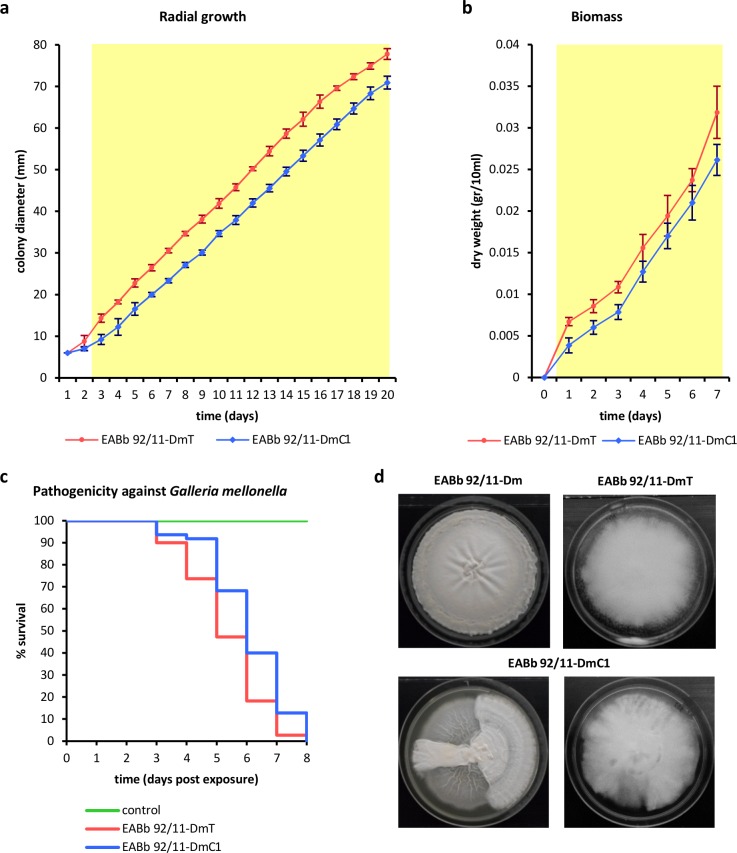
Comparison of the *Beauveria bassiana* EABb 92/11-DmT and EABb 92/11-DmC; virus-transfected and virus-free isogenic lines. Comparison of the fungal growth in solid and liquid medium; **(a)** radial growth and **(b)** biomass production of EABb 92/11-DmC and EABb 92/11-DmT. At least three independent repetitions were performed in triplicate (radial growth) or duplicate (biomass production) and error bars represent standard deviation. A yellow background indicates the time points when the difference in growth is statistically significant (Student’s t test, P-value < 0.05). **(c)** Mean survival curves of *G*. *mellonella* larvae infected with the EABb 92/11-DmC and EABb 92/11-DmT isogenic lines. At least three independent repetitions were performed in triplicate. **(d)** Colonies of the EABb 92/11-Dm, EABb 92/11-DmC and EABb 92/11-DmT grown on Czapek-Dox CM.

Subsequently, attempts were made to reintroduce by transfection purified BbPmV-1 and BbNV-1 into EABb 92/11-DmC1 and in parallel into *B*. *bassiana* ATCC 704040 protoplasts. *B*. *bassiana* ATCC 704040 is a commercially available strain used as biocontrol agent against a variety of arthropod pests, including various flies, thrips, mites, aphids and tingids. *B*. *bassiana* ATCC 704040 is virus-free and, unlike EABb 92/11-DmC (Fig 3B and 3C), was unable to support replication of either BbPmV-1 or BbNV-1, indicating a possible incompatibility between specific mycoviruses and different fungal strains. Similarly, the radial growth, biomass and virulence of the isolates EABb 92/11-DmT and EABb 92/11-DmC1 were compared and small but statistically significant increases in radial growth and biomass production, respectively, were observed for EABb 92/11-DmT in comparison to EABb 92/11-DmC (Student’s t test, P-value < 0.05; Fig 3A and 3B respectively). In particular, a two-day delay in the radial growth of EABb 92/11-DmC in comparison to EABb 92/11-DmT was noted. Additionally, a similar small, but statistically significant decrease in the survival rates of the *G*. *mellonella* larvae infected with EABb 92/11-DmT in comparison to EABb 92/11-DmC was noted (Log-rank test, P-value < 0.05; Wilcoxon test, P-value < 0.05; Fig 3C).

Similar mild hypervirulent effects have been reported for AfuPmV-1 [6] and for another polymycovirus isolated from the *Aspergillus fumigatus* A78 strain [30]; although in the present case it is not known whether the effects are due to BbPmV-1, BbNV-1 or their combination. Since BbNV-1 and BbPmV-1 are both non-conventionally encapsidated viruses as shown by atomic force microscopy (S5F Fig), their separation as distinct particles using sucrose or caesium chloride gradients was not feasible. In terms of phenotype, no significant differences between the isogenic lines were observed apart from frequent sectoring in EABb 92/11-DmC (Fig 3D) as noted for *A*. *fumigatus* [6]. Both sectors are virus-free and the morphology of the low-density sector is transient, since after sub-culture it reverts back to a high-density and cotton-like texture phenotype. This is the first report of a hypervirulent agent found in an entomopathogenic fungus and signifies an important discovery in the field of biological control. Thus mycoviruses might be utilised as enhancers of extant commercially available *B*. *bassiana* strains, an application that constitutes a viable alternative to the genetic engineering of fungal biocontrol agents in order to improve their efficacy against insect pests.

### Mycovirus physiology and life cycle

The quantities of BbPV-1, BbPV-2, BbVV-3, BbPmV-1 and BbNV-1 dsRNAs were assessed in relation to the developmental stages of the fungus. In the case of BbPV-1, BbPV-2 and BbVV-3 (S8A–S8C Fig), a strong negative correlation (PCC = -0.86, -0.78 and -0.99, respectively) between the quantity of dsRNA and the developmental stages of the fungus was noted; more specifically, the dsRNA copy number appears to increase during the early time-points of fungal growth in comparison to later time-points. Additionally, at early time-points, viral dsRNA and proteins were found in the culture supernatant. In contrast, the levels of BbPmV-1 and BbNV-1 dsRNA seem unaffected by the developmental stage of isolate EABb 92/11-Dm and remain stable during all time-points (S8D Fig) and no viral dsRNA was detected in the culture supernatant at any time-point examined. This observation may reflect differences in the replication cycles of encapsidated BbPV-1, BbPV-2 and BbVV-3 and the non-encapsidated BbPmV-1 and BbNV-1.

In conclusion, the present work represents the first comprehensive study of the virome of *Beauveria bassiana*, an ecologically and economically important entomopathogenic ascomycete. New members of already established virus families have been discovered together with the smallest virus to date and a novel mycovirus family that demonstrates an unprecedented dynamic nature in terms of genomic segment number and sequence. Finally, the potential use of exemplar mycoviruses in the biological control of arthropods is highlighted here for the first time and may have significant ecological and economic implications in the future.

## Materials and Methods

### Fungal strains and culture media

All *Beauveria bassiana* isolates used in the study belong to a collection of entomopathogenic fungi maintained in the Department of Genetics and Biotechnology, University of Athens, Greece [11]. All strains were grown on liquid or solid Czapek-Dox complete medium (CM) at 25°C. Conidiospores were harvested from Czapek-Dox CM agar as described [6]. Isolate EABb 92/11-Dm was grown on Czapek-Dox CM agar containing 75–150 mM cycloheximide for two weeks. Subsequently, single conidia were recovered on Czapek-Dox CM agar without cycloheximide and were assessed for the presence of the viruses by northern blotting and RT-PCR amplification. Preparation of protoplasts and transfection were performed as described previously [6].

### Growth and virulence assays

Fungal spores (n = 10^8^) derived from isolates IMI 331273, IMI 392612, EABb 01/103Su and EABb 92/11-Dm were inoculated into 10 mL of Czapek-Dox CM broth and incubated on a rotary shaker (150 rpm) over a period of 7 days. The mycelium from individual cultures was harvested daily by filtration through Miracloth and the pellets were lyophilized before been weighed. In order to assess the viral genome levels, dsRNA was extracted and quantified on an agarose gel using ImageJ [31]. The Pearson’s Correlation Coefficient (PCC) was used to measure the correlation between the fungal biomass and the viral genomic dsRNA. To assess radial growth equal numbers of spores (n = 100) of isogenic lines of virus-infected and virus-free EABb 92/11-Dm strain were centrally inoculated onto Czapek-Dox CM agar and the colony diameters of the isolates were measured every 24 h over a period of 20 days. To assess biomass production equal numbers of spores (n = 10^8^) of the two isolates were inoculated into 10 mL of Czapek-Dox CM broth as described above. *G*. *mellonella* infectivity assays were performed as described [29]. Briefly, 5th-instar *G*. *mellonella* larvae were dipped into 2 mL of 10^5^ spore suspensions for 10 sec. Mortality was recorded every 24 h over a period of 8 days. Log-Rank and Wilcoxon tests as implemented by GraphPad Prism 6 were used to analyse the data for significant differences in survival.

### Nucleic acid extraction and digestion

Mycelia were grown in Czapek-Dox CM broth as above and total RNA samples were prepared using an RNeasy mini kit (Qiagen). LiCl fractionation of dsRNA was carried out as described [32]. Isolation of RNA from purified AfuPmV-1 was performed using phenol/chloroform treatment. DNase I (Promega), RNase A (Sigma), S1 nuclease (Promega) and RNase III (New England Biolabs) treatments of purified dsRNAs were performed according to the manufacturer’s instructions.

### Northern hybridization analyses

Gel electrophoresis, denaturation, neutralization and electrophoretic blotting of dsRNA or ssRNA were carried out according to standard protocols [33]. Blots were hybridized with strand specific riboprobes synthesized by *in vitro* transcription of template DNA in the presence of digoxigenin-UTP, using T7 RNA polymerase (DIG Northern Starter Kit; Roche), followed by immunological detection using alkaline phosphatase-conjugated, anti-digoxigenin antibody (Roche).

### Molecular cloning and RT-qPCR

After electrophoretic separation on agarose gels dsRNAs were used, either collectively or individually, as templates for cDNA synthesis and PCR amplification of products using random priming, sequence-specific priming and RNA ligase-mediated rapid amplification of cDNA ends (RLM-RACE) which were subsequently cloned and sequenced as described [34,35]. At least three different clones were sequenced covering the same part of each viral genome. Determination of the 5’-capped status of the BbPmV-1 and BbNV-1 dsRNAs was conducted using the Ambion First Choice RLM-RACE kit as described [6]. The Real-Time qPCR assays were performed in the OneStepPlus Real-Time qPCR System (Applied Biosystems) using the Power SYBR Green PCR Master Mix (Applied Biosystems) and the relative standard curve quantitation method. BbSNLV-SR and the *B*. *bassiana* ITS sequence that served as an endogenous control were amplified using the target-specific primer pairs 5’-TTC CGC CTC TCG AAT AGA AA-3’ and 5’-CGA ACA GAG TGG CAA GAT GA-3’; 5’-GAT CTC TTG GCT CTG GCA TC-3’ and 5’-TTG AAA TGA CGC TCG AAC AG-3’, respectively, after cDNA synthesis using the same target-specific primers as appropriate.

### Sequence and phylogenetic analyses

Sequence similarity searches of the GenBank, Swissprot and EMBL databases were conducted using the BLAST program [36]. Searches for protein motifs were conducted using the Pfam [37] and CDD [38] databases, and PONDR-FIT [39] was used for the prediction of intrinsically disordered regions. For phylogenetic analysis the protein sequences were aligned with MUSCLE as implemented by MEGA 6 [40], the alignment was improved manually and all positions with less than 30% site coverage were eliminated. Maximum likelihood phylogenetic trees were constructed using MEGA 6 and their topology was confirmed using PhyML [41].

### Mycovirus purification and visualisation

Purification of virus-like particles was performed as described [6]. Purified viruses were negatively stained with 1% uranyl acetate on carbon-coated 400-mesh copper grids and examined in a transmission electron microscope (Zeiss LEO 906E), or examined on poly-L-lysine coated mica in an atomic force microscope MFP-3D-BIO (Asylum Research Inc. an Oxford Instruments Company, Santa Barbara, CA, USA). Mycelium grown on solid Czapek-Dox CM was cut into small pieces and fixed as described [42]. Thin resin sections were stained with uranyl acetate and lead citrate and examined in a Philips 300 transmission electron microscope (Philips, Eindhoven, The Netherlands).

### Protoplast preparation and transfection

Protoplasts of *B*. *bassiana* strains were generated from hyphae using a similar procedure to that described previously [6]. Briefly, the cell walls of fresh mycelia were digested using 3.2% (^w^/_v_) *Trichoderma harzianum* lysing enzyme (Glucanex, Sigma, L-1412), the protoplasts were washed repeatedly in isotonic buffer and the viruses (10 μl at 100 ng/μl × 10^7^ protoplasts) were introduced *via* polyethylene glycol-mediated transfection. The transfected protoplasts were then rescued and regenerated on 1% (^w^/_v_) D-glucose-enriched Czapek-Dox CM agar where they formed a lawn. Pooled transfected fungal colonies were collected and the mycelia was subcultured at least three times. To verify that the mycelia were transfected with BbPmV-1 and BbNV-1, total RNA extracts were prepared from the cultures using the RNeasy Plant Mini Kit (Qiagen) and RT-PCR amplification of viral amplicons 838 and 502 bp in size using gene-specific primer pairs that were designed based on the sequences of BbPmV-1 and BbNV-1, respectively, was performed.

Data deposition: The sequences reported in this paper have been deposited in the GenBank database: Beauveria bassiana partitivirus-1 and -2 (BbPV-1 and -2) accession numbers LN896303-LN896306; Beauveria bassiana victorivirus-1 (BbVV-1) strains EABb 01/103Su, EABb 01/12Su, EABb 00/88Su, EABb 00/23Su accession numbers LN896314-LN896317; Beauveria bassiana polymycovirus-1, -2 and -3 (BbPmV-1, -2 and -3) accession numbers LN896307-LN896313, LN896318-LN896320; Beauveria bassiana small Narna-like virus (BbSNLV) and satellite RNAs accession numbers LT627647, LN896321- LN896322.

## Supporting Information

S1 Table Arthropod host and geographic origin of *Beauveria bassiana* isolates harboring dsRNA elements. (PDF)

S2 Table Taxonomy, acronym and accession number of viruses used for phylogenetic analysis. (PDF)

S3 Table Properties of known polymycoviruses. (PDF)

S1 Fig Population study of dsRNA elements in *Beauveria bassiana*. **(a)** Schematic representation of the electrophoretic profiles of dsRNA elements extracted from 17 *B*. *bassiana* isolates and their relative sizes. **(b)** Geographical distribution of mycoviruses found in *B*. *bassiana*.
(PDF)

S2 Fig Partitiviruses in *Beauveria bassiana*. **(a)** 1% (^w^/_v_) agarose gel electrophoresis of viral dsRNA extracted from *B*. *bassiana* isolates IMI 331273 (lane 2) and IMI 392612 (lane 3), harboring BbPV-1 and BbPV-2, respectively. Lane 1 contains the DNA marker Hyperladder I (Bioline), the sizes of which are shown to the left of the gel. **(b)** TEM images of BbPV-1 in fungal mycelia (left) and negative stained, purified BbPV-2 (right). Both are visualized as icosahedral particles approximately 50 nm in diameter. Virus particles are indicated by arrows. **(c)** Schematic representation of the genomic organisation of BbPV-1 and BbPV-2. The BbPV-1 and BbPV-2 genomes consist of two dsRNAs each containing one ORF (grey boxes) flanked by 5’- and 3’-UTRs (black boxes). **(d)** A comparison of the conserved motifs of the RdRP in BbPV-1, BbPV-2 and other partitiviruses (S2 Table). Numbers within the brackets indicate the number of aa not shown. Asterisks signify identical aa residues, colons signify highly conserved residues and single dots signify less conserved but related residues. **(e)** Maximum likelihood phylogenetic tree created based on the alignment of RdRP and CP sequences of members of the *Partitiviridae* family (S2 Table) using the LG+G+I substitution model. Branches with bootstrap support lower that 50% were collapsed. At the end of the branches, red circles indicate that the virus infects fungi and green circles indicate that the virus infects plants. BbPV-1 and BbPV-2 are indicated by arrows. **(f)** Electrophoresis and northern blot hybridization of *B*. *bassiana* partitiviruses. DsRNA extracted from seven *B*. *bassiana* isolates, including IMI 331273 that harbors BbPV-1 and IMI 392612 that harbors BbPV-2, was electrophoresed in 1% (^w^/_v_) agarose gels and blotted onto nylon membranes. Hybridization was carried out using probes specific for the RdRP and the CP of BbPV-1 and BbPV-2.
(PDF)

S3 Fig Victoriviruses in *Beauveria bassiana*. **(a)** 1% (^w^/_v_) agarose gel electrophoresis of dsRNA extracted from *B*. *bassiana* isolate EABb 01/103Su harboring BbVV-3 using virus purification (lane 3). Lane 1 contains the DNA marker Hyperladder I (Bioline), the sizes of which are shown to the left of the gel. **(b)** Schematic representation of the genomic organisation of BbVV-1. The BbVV-1 genome consists of a single dsRNA that contains two overlapping ORFs encoding a CP (light grey box) and an RdRP (dark grey box) flanked by 5’- and 3’-UTRs (black boxes). In the alignment of partial RdRP sequences of members of the genus *Victorivirus*, family *Totiviridae* isolated from *B*. *bassiana* strains, including BbVV-1 (accession number HE572591; Herrero et al. 2012) and BbVV-2 (accession number NC_024151; Yie et al. 2014), asterisks signify identical aa residues, colons signify highly conserved residues and single dots signify less conserved but related residues.
(PDF)

S4 Fig Sequence properties of known polymycoviruses. Schematic representation of the genomic organisation of **(a)** BdPmV-1 and **(b)** CcPmV-1. Each genome consist of five dsRNAs, each containing one ORF (grey boxes) flanked by 5’- and 3’-UTRs (black boxes). A comparative alignment of the 5’- and 3’-terminal sequences of **(c)** BdPmV-1 dsRNAs 1–5, **(d)** CcPmV-1 dsRNAs 1–5, **(e)** AltPmV-1 dsRNAs 2, 5 and 6, **(f)** BbPmV-1 dsRNAs 1–4 and **(g)** BbPmV-1 dsRNAs 1, 6 and 7. Asterisks signify identical nucleotides. AltPmV-1 dsRNA 6 does not share a similar 3’-terminal sequence with dsRNAs 2 or 5; however there is an internal sequence in its 3’-UTR with significant similarity to the 3’-terminus of dsRNAs 2 and 5, indicating a possible sequencing error. Virus names, acronyms, and GenBank accession numbers are listed in S3 Table.
(PDF)

S5 Fig Sequence and functional analysis of the polymycovirus-encoded proteins. **(a)** Comparison of the conserved motifs of the RdRP in polymycoviruses. Numbers within the brackets indicate the number of aa not shown. In the RdRP motifs, the symbol ‘#’ signifies S or T and the symbol ‘&’ signifies bulky hydrophobic residues (I, L, V, M, F, Y, W). In all sequence alignments, asterisks signify identical aa residues, colons signify highly conserved residues and single dots signify less conserved but related residues. **(b)** Comparison of the cysteine-rich zinc finger-like found in the proteins encoded by dsRNA 2 of polymycoviruses. **(c)** Comparison of the catalytic methyltransferase motifs found in the proteins encoded by dsRNA 3 of polymycoviruses and 5’-RLM RACE oligo-cap analysis of BbPmV-1 dsRNA 1. Viral dsRNA was (i) treated with CIP and TAP and ligated to an oligoribonucleotide adaptor at the 5’-terminus, (ii) treated with CIP only and ligated, (iii) left untreated and ligated and (iv) left untreated and unligated. Subsequently, RT-PCR was performed with a primer homologous to the oligonucleotide adaptor and a sequence-specific primer, and the products were electrophoresed on a 4.5% (^w^/_v_) native polyacrylamide gel. The expected product size is 310 bp, indicated by an arrow, and the positive reaction in lane 1 indicates that the positive-strand of dsRNA 1 is capped. **(d)** Proline-alanine-serine (PAS) content of the proteins encoded by RNA 4 of polymycoviruses. **(e)** PAS content of the proteins encoded by ORF-1 of unirnaviruses. **(f)** AFM image of the non-conventionally encapsidated viruses from the EABb 92/11-Dm isolate. BbPmV-1 and BbNV-1 are visualized as chain-like linear nucleic acids of different lengths corresponding to those predicted from the size of the genomic dsRNAs. **(g)** PAS content of the proteins encoded by the extra dsRNAs of polymycoviruses. Virus names, acronyms, and GenBank accession numbers are listed in S3 Table.
(PDF)

S6 Fig Phylogenetic analysis of polymycoviruses. Maximum likelihood phylogenetic tree created based on the alignment of RdRP sequences of polymycoviruses and related viruses belonging to the Superfamily 1 (S2 and S3 Tables) using the rtREV+G+I+F substitution model. Branches with bootstrap support lower that 50% were collapsed. At the end of the branches, circles indicate that the virus has a dsRNA genome and squares indicate that the virus has a ssRNA genome. Red, green and blue indicate that the virus infects fungi, plants and vertebrates respectively. Next to the virus family name the presence of a hexagon indicates that members of the family are known to be conventionally encapsidated. BbPmV-1 and BbPmV-2 are indicated by arrows.
(PDF)

S7 Fig Comparison of the *Beauveria bassiana* EABb 92/11-Dm and EABb 92/11-DmC; virus-infected and virus-free isogenic lines. **(a)** 1% (^w^/_v_) agarose gel electrophoresis and northern blot hybridization of dsRNA extracted from the EABb 92/11-Dm and EABb 92/11-DmC1 isogenic lines. Hybridization was carried out using a probe specific for BbPmV-1 dsRNA1. Attempted RT-PCR amplification of **(b)** a 838 bp BbPmV-1 dsRNA1 fragment and **(c)** a 502 bp BbNV-1 dsRNA fragment from the EABb 92/11-Dm, EABb 92/11-DmC1, EABb 92/11-DmC2, EABb 92/11-DmC3 and EABb 92/11-DmT isogenic lines. In all gels, lane 1 contains the DNA marker Hyperladder I (Bioline), the sizes of which are shown to the left of the gel. Comparison of the fungal growth in solid and liquid medium; **(d)** radial growth and **(e)** biomass production of EABb 92/11-Dm, EABb 92/11-DmC1, EABb 92/11-DmC2 and EABb 92/11-DmC3. At least three independent repetitions were performed in triplicate (radial growth) or duplicate (biomass production) and error bars represent standard deviation. A yellow background indicates the time points when the difference in growth is statistically significant (Student’s t test, P-value < 0.05). **(f)** Mean survival curves of *G*. *mellonella* larvae infected with the EABb 92/11-Dm, EABb 92/11-DmC1, EABb 92/11-DmC2 and EABb 92/11-DmC3 isogenic lines. At least three independent repetitions were performed in triplicate.
(PDF)

S8 Fig Time course study of viral dsRNA levels correlated with fungal growth. Biomass production of isolates **(a)** IMI 331273, **(b)** IMI 392612, **(c)** EABb 01/103Su and **(d)** EABb 92/11-Dm in liquid Czapek-Dox CM was assessed daily for 7 days (grey shading). **(a)** BbPV-1, **(b)** BbPV-2, **(c)** BbVV-3, **(d)** BbNV-1 and BbPmV-1 dsRNAs extracted from equal amounts of dry mycelia were electrophoresed in 1% (^w^/_v_) agarose gels, dsRNA levels were quantified by ImageJ and the results are presented in graphical form (black lines). At least three independent repetitions were performed in duplicate and error bars represent standard deviation. Representative agarose gel analyses of the dsRNAs are shown below each graph.
(PDF)
