# Supplementary material for: Studies on the Virome of the Entomopathogenic Fungus Beauveria bassiana Reveal Novel dsRNA Elements and Mild Hypervirulence
Source: PLoS Pathog. 2017 Jan 23;13(1):e1006183. doi: 10.1371/journal.ppat.1006183 (PMC5293280; doi:10.1371/journal.ppat.1006183)
Supplement: S1 Table — (PDF) [file ppat.1006183.s001.pdf]

S1 Table. Arthropod host and geographic origin of *B. bassiana* isolates harboring dsRNA elements.

***B. bassiana* isolate**	**Arthropod Host**				**Location**
	Species/Genus	Family	Superfamily	Order	
IMI 392612	Adult spider	*Araneidae*	*Araneoidea*	*Arachnida*	Ecuador
IMI 386705	*Diabrotica speciosa*	*Chryssomelidae*	*Chrysomeloidea*	*Coleoptera*	Brazil
IMI 331273 (ARSEF 757)	Terebrinoid beetle	*Terebrionidae*	*Terebrionoidea*		Brazil
IMI 391043	*Eurygaster sp.*		*Pentatomoidea*	*Hemiptera*	Syria
IMI 391044	*Eurygaster integriceps*		*Pentatomoidea*		Syria
IMI 391362	*Eurygaster integriceps*		*Pentatomoidea*		Syria
IMI 391704	*Eurygaster integriceps*		*Pentatomoidea*		Syria
SP R 159	*Eurygaster integriceps*		*Pentatomoidea*		Russia
SP R 184	*Eurygaster integriceps*		*Pentatomoidea*		Russia
SP U 259	*Eurygaster integriceps*		*Pentatomoidea*		Uzbekistan
EABb 92/11-Dm	*Dociostaurus maroccanus*	*Acrididae*	*Acridoidea*	*Orthoptera*	Spain "la Serena" Badajoz
ATHUM 4946	Air-borne				Greece, Athens
EABb 01/103Su	Soil, forest				Spain, Seville
EABb 01/12Su	Soil, non-cultivated area				
EABb 01/88Su	Soil, sunflower				Portugal (south)
EABb 00/23Su	Soil, pasture				Canary Islands (Tenerife)
