# Supplementary material for: Studies on the Virome of the Entomopathogenic Fungus Beauveria bassiana Reveal Novel dsRNA Elements and Mild Hypervirulence
Source: PLoS Pathog. 2017 Jan 23;13(1):e1006183. doi: 10.1371/journal.ppat.1006183 (PMC5293280; doi:10.1371/journal.ppat.1006183)
Supplement: S2 Table — (PDF) [file ppat.1006183.s002.pdf]

S2 Table. Taxonomy, acronym and accession number of viruses used for phylogenetic analysis.

Family	Genus	Virus name	Acronym	Accession number		Reference
				RdRP	CP	
Amalgaviridae	*Amalgavirus*	Blueberry latent virus	BLV	YP_003934623		1
		Rhododendron virus A	RV-A	YP_003868436		2
		Southern tomato virus	STV	YP_002321509		3
		Vicia cryptic virus M	VCV-M	EU371896		unpublished
Astroviridae	*Avastrovirus*	Avastrovirus 1	AAV-1	CAB95006		4
		Avastrovirus 3	AAV-3	YP_002728002		5
	Mamastrovirus	Mamastrovirus 1	MAV-1	NP_059443		6
		Mamastrovirus 10	MAV-10	AAO32082		7
Caliciviridae	*Lagovirus*	Rabbit haemorrhagic disease virus	RHCV	NP_062875		8
	Nebovirus	Newbury-1 virus	NB-1V	NP_663315		9
	Norovirus	Norwalk virus	NV	NP_056820		10
	Sapovirus	Sapporo virus	SV	NP_051035		11
	Vesivirus	Vesicular exanthema of swine virus	VESV	NP_786896		12
Partitiviridae	*Alphapartitivirus*	White clover cryptic virus 1	WCCC-1	YP_086754	YP_086755	13
	Betapartitivirus	Atkinsonella hypoxylon partitivirus	AhPV	NP_604475	NP_604476	14
	Gammapartitivirus	Aspergillus fumigatus partitivirus-1	AfuPV-1	CAY25801	CAZ61323	15
		Aspergillus ochraceous virus	AoV	ABV30675	ABV30676	16
		Beauveria bassiana partitivirus-1	BbPV-1	LN896303	LN896304	
		Beauveria bassiana partitivirus-2	BbPV-2	LN896305	LN896306	
		Botryotinia fuckeliana partitivirus 1	BfPV-1	CAM33266	CAM33267	unpublished
		Discula destructiva virus 1	DdV-1	AAG59816	AAK13165	17
		Discula destructiva virus 2	DdV-2	AAK59379	AAK59380	17
		Fusarium solani virus 1	FsV-1	BAA09520	BAA09521	18
		Gremmeniella abietina RNA virus MS1	GaV-MS1	AAM12240	AAM12241	19
		Ophiostoma partitivirus 1	OPV-1	CAJ31886	CAJ31887	20
		Penicillium stoloniferum virus F	PsV-F	AAU95758	AAU95759	21
		Penicillium stoloniferum virus S	PsV-S	YP_052856	YP_052857	22
		Ustilaginoidea virens partitivirus	UvPV	AGO04402	AGO04403	23
		Verticillium dahliae partitivirus 1	VdPV-1	AGI52210	AGI52209	24
	Deltapartitivirus	Beet cryptic virus 2	BCV-1	ADP24757		unpublished
		Beet cryptic virus 3	BCV-3	AAB27624		25
		Fig cryptic virus	FCV	YP_004429258		26
		Pepper cryptic virus 1	PCV-1	AEJ07890	AEJ07891	27
		Pepper cryptic virus 2	PCV-2	AEJ07892		27
Narnaviridae	*Mitovirus*	Ophiostoma mitovirus 4	OMV-4	NP_660179		28
	Narnavirus	Beauveria bassiana small Narna-like virus	BbSNLV	LT627647		
		Leptomonas seymouri Narna-like virus 1	LsNLV-1	ANI86034		unpublished
		Phytomonas serpens narnavirus 1	PsNV-1	YP_009256557		29
		Phytophthora infestans RNA virus 4	PiRV-4	AEM89293		30
		Saccharomyces cerevisiae 20S RNA narnavirus	ScNV-20S	NP_660178		31
		Saccharomyces cerevisiae 23S RNA narnavirus	ScNV-23S	NP_660177		31
Unirnaviridae*	Unirnavirus	Alternaria longipes dsRNA virus 1	AlRV-1	AIJ01443		32
		Beauveria bassiana non-segmented virus 1	BbNV-1	LN610699		33
		Beauveria bassiana RNA virus 1	BbRV-1	AKC57301		34
		Ustilaginoidea virens unassigned RNA virus HNND-1	UvRV-HNND-1	YP_009154709		35
unassigned		Cryphonectria parasitica bipartite mycovirus 1	CpV-1	YP_007985675		unpublished
		Fusarium graminearum dsRNA mycovirus-4	FgV-4	YP_003288790		36
		Gremmeniella abietina RNA virus 6	GaRV-6	AIU98624		unpublished
		Heterobasidion RNA virus 6	HRV-6	AHA82547		37
		Rhizoctonia fumigata mycovirus	RfuV	YP_009134757		unpublished
		Rhizoctonia solani dsRNA virus 1	RsRV-1	AFZ85210		38

*proposed family

1. Martin RR, Zhou J and Tzanetakis IE (2011) Blueberry latent virus: An amalgam of the *Partitiviridae* and *Totiviridae*. *Virus Res* 155(1):175-180.
2. Sabanadzovic S, Abou Ghanem-Sabanadzovic N and Valverde RA (2010) A novel monopartite dsRNA virus from rhododendron. *Arch Virol* 155(11):1859-1863.
3. Sabanadzovic S, Valverde RA, Brown JK, Martin RR and Tzanetakis IE (2009) Southern tomato virus: The link between the families *Totiviridae* and *Partitiviridae*. *Virus Res* 140(1-2):130-137.
4. Jonassen CM, Jonassen TO, Sveen TM and Grinde B (2003) Complete genomic sequences of astroviruses from sheep and turkey: comparison with related viruses. *Virus Res* 91(2):195-201.
5. Fu Y, Pan M, Wang X, Xu Y, Xie X, Knowles NJ, Yang H and Zhang D (2009) Complete sequence of a duck astrovirus associated with fatal hepatitis in ducklings. *J Gen Virol* 90(Pt 5): 1104-1108.
6. Willcocks MM, Brown TD, Madeley CR and Carter MJ (1994) The complete sequence of a human astrovirus. *J Gen Virol* 75(Pt 7):1785-1788.
7. Mittelholzer C, Hedlund KO, Englund L, Dietz HH and Svensson L (2003) Molecular characterization of a novel astrovirus associated with disease in mink. *J Gen Virol* 84(Pt 11):3087-3094.
8. Meyers G, Wirblich C and Thiel HJ (1991) Rabbit hemorrhagic disease virus--molecular cloning and nucleotide sequencing of a calicivirus genome. *Virology* 184(2):664-676.
9. Smiley JR, Chang KO, Hayes J, Vinje J and Saif LJ (2002) Characterization of an enteropathogenic bovine calicivirus representing a potentially new calicivirus genus. *J Virol* 76(20):10089-10098.
10. Liu B, Clarke IN and Lambden PR (1996) Polyprotein processing in Southampton virus: identification of 3C-like protease cleavage sites by *in vitro* mutagenesis. *J Virol* 70(4):2605-2610.
11. Guo M, Chang KO, Hardy ME, Zhang Q, Parwani AV and Saif LJ (1999) Molecular characterization of a porcine enteric calicivirus genetically related to Sapporo-like human caliciviruses. *J Virol* 73(11):9625-9631.
12. Neill JD, Meyer RF and Seal BS (1998) The capsid protein of vesicular exanthema of swine virus serotype A48: relationship to the capsid protein of other animal caliciviruses. *Virus Res* 54(1):39-50.
13. Boccardo G and Candresse T (2005) Complete sequence of the RNA1 of an isolate of White clover cryptic virus 1, type species of the genus *Alphacryptovirus*. *Arch Virol* 150(2):399-402.
14. Oh CS and Hillman BI (1995) Genome organization of a partitivirus from the filamentous ascomycete *Atkinsonella hypoxylon*. *J Gen Virol* 76(Pt 6): 1461-1470.
15. Bhatti MF, Bignell EM and Coutts RHA (2011) Complete nucleotide sequences of two dsRNAs associated with a new partitivirus infecting *Aspergillus fumigatus*. *Arch Virol* 156(9):1677-1680.
16. Liu W, Duns G and Chen J (2008) Genomic characterization of a novel partitivirus infecting *Aspergillus ochraceus*. *Virus Genes* 37(3):322-327.
17. Rong R, Rao S, Scott SW, Carner GR and Tainter FH (2002) Complete sequence of the genome of two dsRNA viruses from *Discula destructiva*. *Virus Res* 90(1-2):217-224.
18. Nogawa M, Kageyama T, Nakatani A, Taguchi G, Shimosaka M and Okazaki M (1996) Cloning and characterization of mycovirus double-stranded RNA from the plant pathogenic fungus, *Fusarium solani f. sp. Robiniae*. *Biosci Biotechnol Biochem* 60(5):784-788.
19. Tuomivirta TT and Hantula J (2003) Two unrelated double-stranded RNA molecule patterns in *Gremmeniella abietina* type A code for putative viruses of the families *Totiviridae* and *Partitiviridae*. *Arch Virol* 148(12):2293-2305.
20. Crawford LJ, Osman TA, Booy FP, Coutts RHA, Brasier CM and Buck KW (2006) Molecular characterization of a partitivirus from *Ophiostoma himal-ulmi*. *Virus Genes* 33(1):33-39.
21. Kim JW, Choi EY and Lee JI (2005) Genome organization and expression of the Penicillium stoloniferum virus F. *Virus Genes* 31(2):175-183.
22. Kim JW, Kim SY and Kim KM (2003) Genome organization and expression of the Penicillium stoloniferum virus S. *Virus Genes* 27(3):249-256.
23. Zhang T, Jiang Y, Huang J and Dong W (2013) Genomic organization of a novel partitivirus from the phytopathogenic fungus *Ustilaginoidea virens*. *Arch Virol* 158(11):2415-2419.

24. Feng Z, Zhu H, Li Z, Shi Y, Zhao L, Liu L and Jiang D (2013) Complete genome sequence of a novel dsRNA mycovirus isolated from the phytopathogenic fungus *Verticillium dahliae Kleb*. *Arch Virol* 158(12):2621-2623.
25. Xie WS, Antoniw JF and White RF (1993) Nucleotide sequence of beet cryptic virus 3 dsRNA2 which encodes a putative RNA-dependent RNA polymerase. *J Gen Virol* 74(Pt 7):1467-1470.
26. Elbeaino T, Kubaa RA, Digiaro M, Minafra A and Martelli GP (2011) The complete nucleotide sequence and genome organization of Fig cryptic virus, a novel bipartite dsRNA virus infecting fig, widely distributed in the Mediterranean basin. *Virus Genes* 42(3):415-421.
27. Sabanadzovic S and Valverde RA (2011) Properties and detection of two cryptoviruses from pepper (*Capsicum annuum*). *Virus Genes* 43(2):307-312.
28. Hong Y, Dover SL, Cole TE, Brasier CM and Buck KW (1999) Multiple mitochondrial viruses in an isolate of the Dutch Elm disease fungus *Ophiostoma novo-ulmi*. *Virology* 258(1):118-127.
29. Akopyants NS, Lye L-F, Dobson DE, Lukes J and Beverley SM (2016) A narnavirus in the trypanosomatid protist plant pathogen *Phytomonas serpens*. *Genome Announc* 4(4):e00711-16.
30. Cai G, Myers K, Fry WE and Hillman BI (2012) A member of the virus family *Narnaviridae* from the plant pathogenic oomycete *Phytophthora infestans*. *Arch Virol* 157(1):165-169.
31. Rodriguez-Cousino N, Solorzano A, Fujimura T and Esteban R (1998) Yeast positive-stranded virus-like RNA replicons. 20 S and 23 S RNA terminal nucleotide sequences and 3' end secondary structures resemble those of RNA coliphages. *J Biol Chem* 273(32), 20363-20371.
32. Lin Y, Zhang H, Zhao C, Liu S and Guo L (2015) The complete genome sequence of a novel mycovirus from *Alternaria longipes* strain HN28. *Arch Virol* 160(2):577-580.
33. Kotta-Loizou I, Sipkova J and Coutts RHA (2015) Identification and sequence determination of a novel double-stranded RNA mycovirus from the entomopathogenic fungus *Beauveria bassiana*. *Arch Virol* 160(3):873-875.
34. Koloniuk I, Hrabakova L and Petrzik K (2015) Molecular characterization of a novel amalgavirus from the entomopathogenic fungus *Beauveria bassiana*. *Arch Virol* 160(6):1585-1588.
35. Zhu HJ, Chen D, Zhong J, Zhang SY and Gao BD (2015) A novel mycovirus identified from the rice false smut fungus *Ustilaginoidea virens*. *Virus Genes* 51(1):159-162.
36. Yu J, Kwon SJ, Lee KM, Son M and Kim KH (2009) Complete nucleotide sequence of double-stranded RNA viruses from *Fusarium graminearum* strain DK3. *Arch Virol* 154(11):1855-1858.
37. Vainio EJ, Muller MM, Korhonen K, Piri T and Hantula J (2015) Viruses accumulate in aging infection centers of a fungal forest pathogen. *ISME J* 9(2):497-507.
38. Zheng L, Liu H, Zhang M, Cao X and Zhou E (2013) The complete genomic sequence of a novel mycovirus from *Rhizoctonia solani* AG-1 IA strain B275. *Arch Virol* 158 (7):1609-1612.
