# Supplementary material for: Studies on the Virome of the Entomopathogenic Fungus Beauveria bassiana Reveal Novel dsRNA Elements and Mild Hypervirulence
Source: PLoS Pathog. 2017 Jan 23;13(1):e1006183. doi: 10.1371/journal.ppat.1006183 (PMC5293280; doi:10.1371/journal.ppat.1006183)
Supplement: S3 Table — (PDF) [file ppat.1006183.s003.pdf]

S3 Table. Properties of known polymycoviruses.

Virus name	Abbreviation	Segment (bp)	Accession number	ORF size (nt; aa; kDa)	UTR length (bp)	
					5′-UTR	3′-UTR
Aspergillus fumigatus polymycovirus-1	AfuPmV-1	dsRNA 1 (2,403)	HG975302	2,292; 763; 84	35	76
		dsRNA 2 (2,233)	HG975303	2,091; 696; 76	70	72
		dsRNA 3 (1,970)	HG975304	1,845; 614; 67	51	74
		dsRNA 4 (1,131)	HG975305	840; 279; 29	86	205
Beauveria bassiana polymycovirus-1	BbPmV-1	dsRNA 1 (2,425)	LN896307	2,328; 775; 86	26	71
		dsRNA 2 (2,260)	LN896308	2,115; 704; 75	71	74
		dsRNA 3 (1,921)	LN896309	1,833; 610; 67	31	57
		dsRNA 4 (1,373)	LN896310	921; 306; 32	72	380
Botryosphaeria dothidea polymycovirus-1 [1]	BdPmV-1	dsRNA 1 (2,379)	KP245734	2,274; 757; 82	53	70
		dsRNA 2 (2,184)	KP245735	2,085; 694; 75	70	29
		dsRNA 3 (1,967)	KP245736	1,719; 572; 62	57	191
		dsRNA 4 (1,131)	KP245737	828; 275; 29	89	214
		dsRNA 5 (1,060)	KP245738	840; 279; 30	98	122
Cladosporium cladosporioides polymycovirus-1	CcPmV-1	dsRNA 1 (2,434)	NC_024704	2,283; 760; 83	43	108
		dsRNA 2 (2,241)	NC_024705	2,046; 681; 73	72	123
		dsRNA 3 (2,008)	NC_024706	1,860; 619; 67	57	91
		dsRNA 4 (1,261)	NC_024707	885; 294; 31	185	191
		dsRNA 5 (942)	NC_024708	720; 239; 26	90	132
Alternaria polymycovirus-1	AltPmV-1	dsRNA 2 (2,233)	FJ595830	2,091; 686; 75	71	71
		dsRNA 5 (1,365)	FJ595831	1,170; 389; 42	97	98
		dsRNA 6 (1,033)	FJ595832	543; 176; 19	119	371
Beauveria bassiana polymycovirus-2	BbPmV-2	dsRNA 1 (2,405)	LN896311	2,304; 767; 83	26	75
		dsRNA 6 (1,009)	LN896312	750; 249; 27	100	159
		dsRNA 7 (868)	LN896313	618; 205; 22	104	146
Melampsora lini polymycovirus-1 [2]	MlPmV-1	dsRNA 3 (1,932)	X64371	1,845; 614; 67	29	58
Alternaria tenuissima polymycovirus-1	AtPmV-1	dsRNA 1 (1,613)*	KP067914			
Beauveria bassiana polymycovirus-3	BbPmV-3	dsRNA 1 (165)*	LN896318			
		dsRNA 2 (443)*	LN896319			
		dsRNA 3 (259)*	LN896320			
Melampsora lini polymycovirus-1	MlPmV-1	dsRNA 2 (1,768)*				

*partial sequence

1. Zhai L, Xiang J, Zhang M, Fu M, Yang Z, Hong N, Wang G (2016) Characterization of a novel double-stranded RNA mycovirus conferring hypovirulence from the phytopathogenic fungus *Botryosphaeria dothidea*. *Virology* 493:75-85.
2. Dickinson MJ, Zhang R and Pryor A (1993) Nucleotide sequence relationships of double-stranded RNAs in flax rust, *Melampsora lini*. *Curr Genet* 24(5):428-432.
