# Supplementary material for: Studies on the Virome of the Entomopathogenic Fungus Beauveria bassiana Reveal Novel dsRNA Elements and Mild Hypervirulence
Source: PLoS Pathog. 2017 Jan 23;13(1):e1006183. doi: 10.1371/journal.ppat.1006183 (PMC5293280; doi:10.1371/journal.ppat.1006183)
Supplement: S1 Fig — (a) Schematic representation of the electrophoretic profiles of dsRNA elements extracted from 17 B. bassiana isolates and their relative sizes. (b) Geographical distribution of mycoviruses found in B. bassiana. (PDF) [file ppat.1006183.s004.pdf]

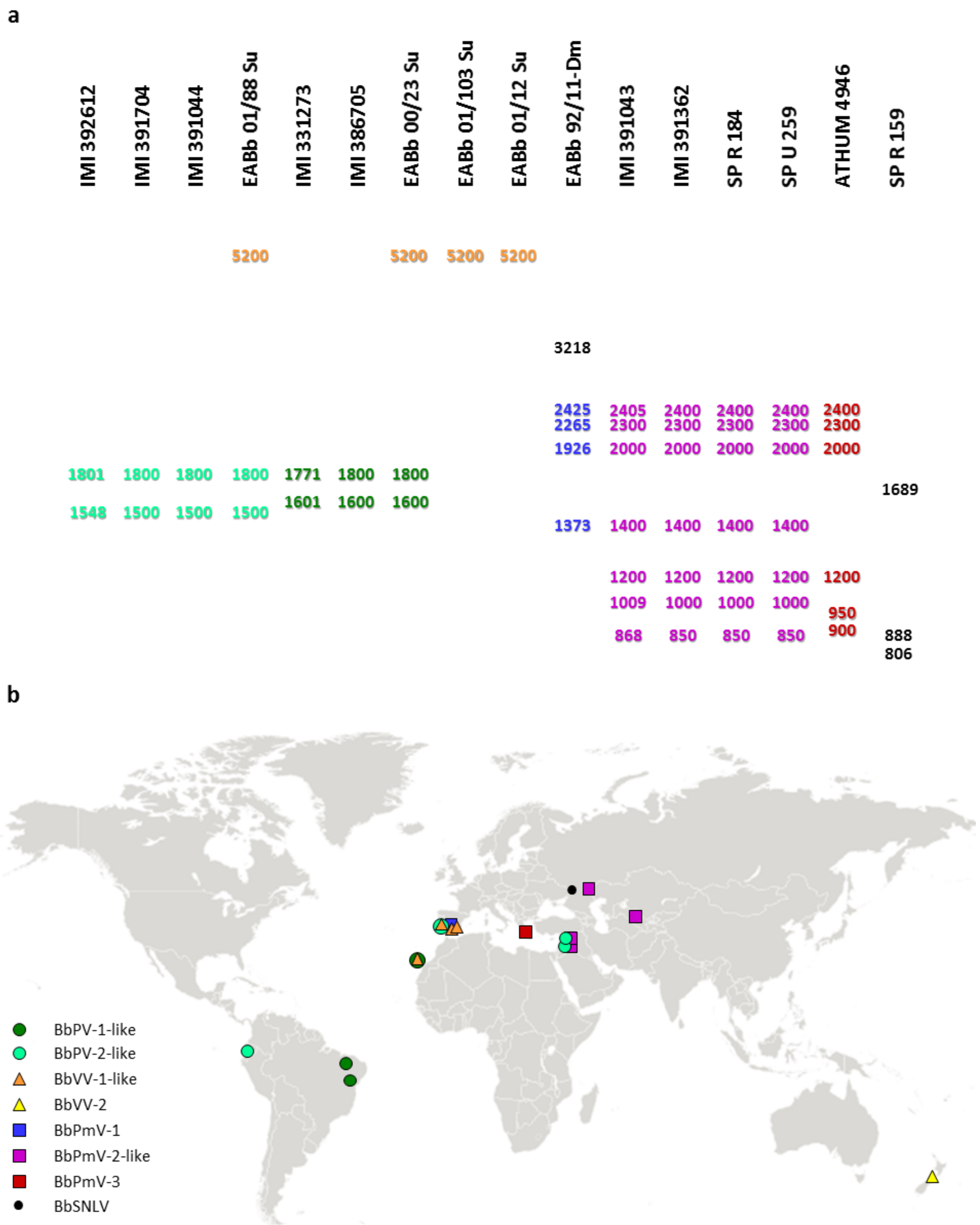


**S1 Fig. Population study of dsRNA elements in *Beauveria bassiana*. (a)** Schematic representation of the electrophoretic profiles of dsRNA elements extracted from 17 *B. bassiana* isolates and their relative sizes. **(b)** Geographical distribution of mycoviruses found in *B. bassiana*.
