# Supplementary material for: Studies on the Virome of the Entomopathogenic Fungus Beauveria bassiana Reveal Novel dsRNA Elements and Mild Hypervirulence
Source: PLoS Pathog. 2017 Jan 23;13(1):e1006183. doi: 10.1371/journal.ppat.1006183 (PMC5293280; doi:10.1371/journal.ppat.1006183)
Supplement: S2 Fig — (a) 1% (w/v) agarose gel electrophoresis of viral dsRNA extracted from B. bassiana isolates IMI 331273 (lane 2) and IMI 392612 (lane 3), harboring BbPV-1 and BbPV-2, respectively. Lane 1 contains the DNA marker Hyperladder I (Bioline), the sizes of which are shown to the left of the gel. (b) TEM images of BbPV-1 in fungal mycelia (left) and negative stained, purified BbPV-2 (right). Both are visualized as icosahedral particles approximately 50 nm in diameter. Virus particles are indicated by arrows. (c) Schematic representation of the genomic organisation of BbPV-1 and BbPV-2. The BbPV-1 and BbPV-2 genomes consist of two dsRNAs each containing one ORF (grey boxes) flanked by 5’- and 3’-UTRs (black boxes). (d) A comparison of the conserved motifs of the RdRP in BbPV-1, BbPV-2 and other partitiviruses (S2 Table). Numbers within the brackets indicate the number of aa not shown. Asterisks signify identical aa residues, colons signify highly conserved residues and single dots signify less conserved but related residues. (e) Maximum likelihood phylogenetic tree created based on the alignment of RdRP and CP sequences of members of the Partitiviridae family (S2 Table) using the LG+G+I substitution model. Branches with bootstrap support lower that 50% were collapsed. At the end of the branches, red circles indicate that the virus infects fungi and green circles indicate that the virus infects plants. BbPV-1 and BbPV-2 are indicated by arrows. (f) Electrophoresis and northern blot hybridization of B. bassiana partitiviruses. DsRNA extracted from seven B. bassiana isolates, including IMI 331273 that harbors BbPV-1 and IMI 392612 that harbors BbPV-2, was electrophoresed in 1% (w/v) agarose gels and blotted onto nylon membranes. Hybridization was carried out using probes specific for the RdRP and the CP of BbPV-1 and BbPV-2. (PDF) [file ppat.1006183.s005.pdf]

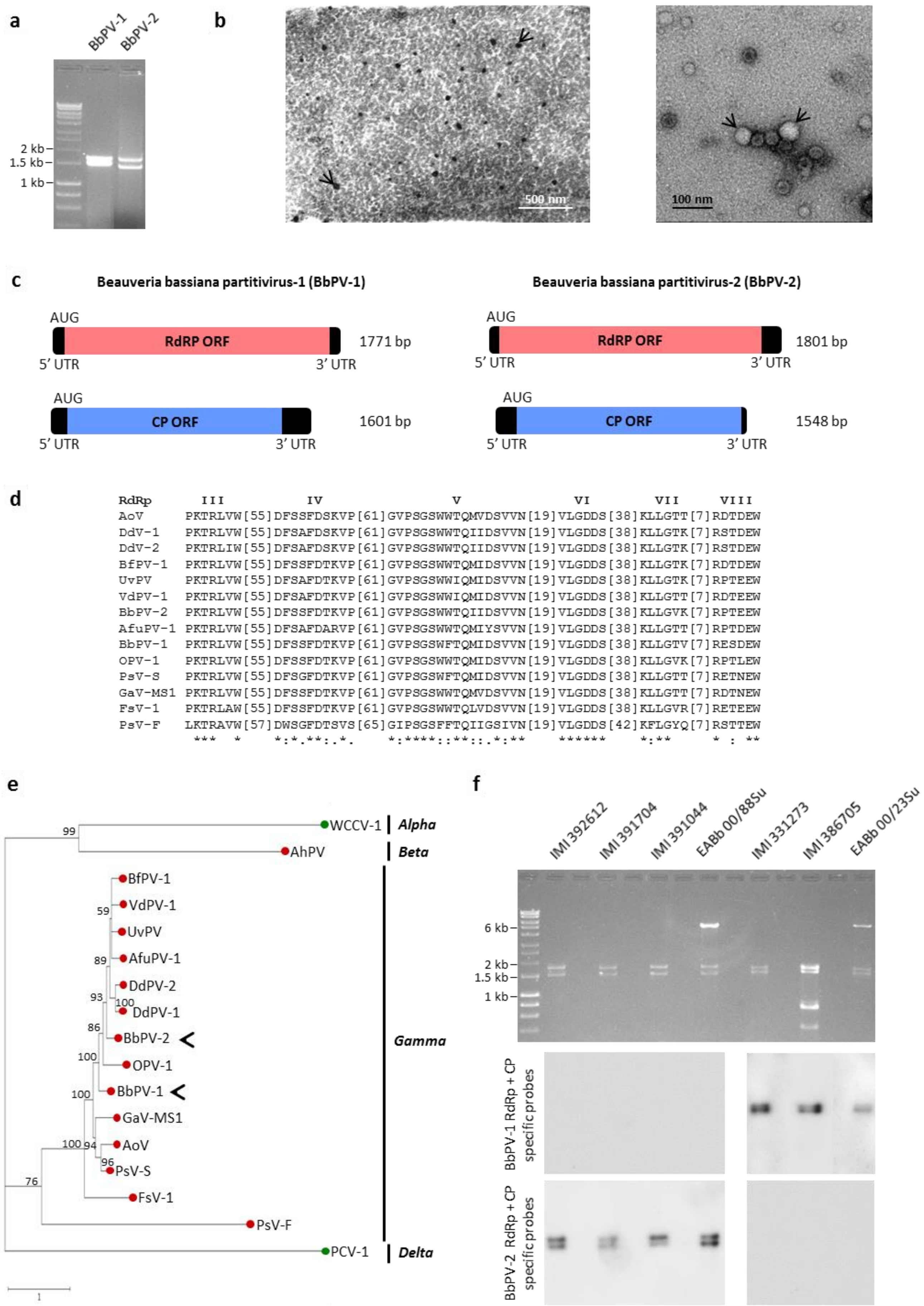

S2 Fig. Partitiviruses in *Beauveria bassiana*. (a) 1% ($^{w}/_{v}$) agarose gel electrophoresis of viral dsRNA extracted from *B. bassiana* isolates IMI 331273 (lane 2) and IMI 392612 (lane 3), harboring BbPV-1 and

BbPV-2, respectively. Lane 1 contains the DNA marker Hyperladder I (Bioline), the sizes of which are shown to the left of the gel. **(b)** TEM images of BbPV-1 in fungal mycelia (left) and negative stained, purified BbPV-2 (right). Both are visualized as icosahedral particles approximately 50 nm in diameter. Virus particles are indicated by arrows. **(c)** Schematic representation of the genomic organisation of BbPV-1 and BbPV-2. The BbPV-1 and BbPV-2 genomes consist of two dsRNAs each containing one ORF (grey boxes) flanked by 5'- and 3'-UTRs (black boxes). **(d)** A comparison of the conserved motifs of the RdRP in BbPV-1, BbPV-2 and other partitiviruses (S2 Table). Numbers within the brackets indicate the number of aa not shown. Asterisks signify identical aa residues, colons signify highly conserved residues and single dots signify less conserved but related residues. **(e)** Maximum likelihood phylogenetic tree created based on the alignment of RdRP and CP sequences of members of the *Partitiviridae* family (S2 Table) using the LG+G+I substitution model. Branches with bootstrap support lower that 50% were collapsed. At the end of the branches, red circles indicate that the virus infects fungi and green circles indicate that the virus infects plants. BbPV-1 and BbPV-2 are indicated by arrows. **(f)** Electrophoresis and northern blot hybridization of *Beauveria bassiana* partitiviruses. DsRNA extracted from seven *B. bassiana* isolates, including IMI 331273 that harbors BbPV-1 and IMI 392612 that harbors BbPV-2, was electrophoresed in 1% ($^{w}/_{v}$) agarose gels and blotted onto nylon membranes. Hybridization was carried out using probes specific for the RdRP and the CP of BbPV-1 and BbPV-2.
