# Supplementary material for: Studies on the Virome of the Entomopathogenic Fungus Beauveria bassiana Reveal Novel dsRNA Elements and Mild Hypervirulence
Source: PLoS Pathog. 2017 Jan 23;13(1):e1006183. doi: 10.1371/journal.ppat.1006183 (PMC5293280; doi:10.1371/journal.ppat.1006183)
Supplement: S3 Fig — (a) 1% (w/v) agarose gel electrophoresis of dsRNA extracted from B. bassiana isolate EABb 01/103Su harboring BbVV-3 using virus purification (lane 3). Lane 1 contains the DNA marker Hyperladder I (Bioline), the sizes of which are shown to the left of the gel. (b) Schematic representation of the genomic organisation of BbVV-1. The BbVV-1 genome consists of a single dsRNA that contains two overlapping ORFs encoding a CP (light grey box) and an RdRP (dark grey box) flanked by 5’- and 3’-UTRs (black boxes). In the alignment of partial RdRP sequences of members of the genus Victorivirus, family Totiviridae isolated from B. bassiana strains, including BbVV-1 (accession number HE572591; Herrero et al. 2012) and BbVV-2 (accession number NC_024151; Yie et al. 2014), asterisks signify identical aa residues, colons signify highly conserved residues and single dots signify less conserved but related residues. (PDF) [file ppat.1006183.s006.pdf]

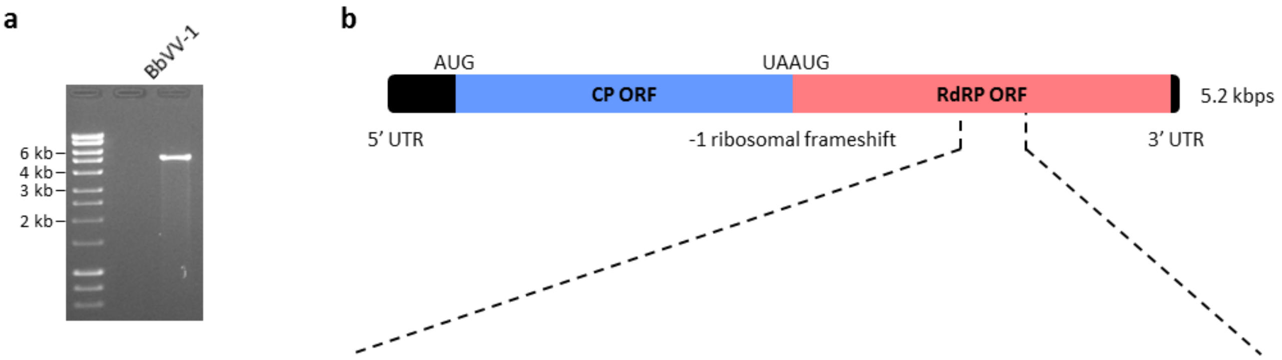


```
BbVV-1 (HE572591)       CDTRSYFAFSWVLGSVQKAWRNSRVILDPGTGGHLGMAQRIINAQRGGGVNLMLDYDDFNSHHSNGVMAMVFD
       EABb 01/103Su    CDTRSYFAFSWVLGSVQKAWRNSRVILDPGTGGHLGMAQRIINSQRGGGVNLMLDYDDFNSHHSNGVMAMVFD
       EABb 01/12Su     CDTRSYFAFSWVLGSVQKAWRNSRVILDPGTGGHLGMAQRIINSQRGGGVNLMLDYDDFNSHHSNGVMAMVFD
       EABb 00/88Su     CDTRSYFAFSWVLGSVQKAWRNSRVILDPGTGGHLGMAQRIINSQRGGGVNLMLDYDDFNSHHSNGVMAMVFD
       EABb 00/23Su     CDTRSYFAFSWVLGSVQKAWRNSRVILDPGTGGHLGMAQRIINSQRGGGVNLMLDYDDFNPHHSNGVMAMVFD
BbVV-2 (NC_024151)      CDTRSYFAFEWILGPVQRSWRNSRVLLDPGFGGHVGIVKKVQALQRGGGVNLMLDYDDFNSAHSTATMQMVFD
                        *********.*:**.**::******:**** ***:*:.:::   ****************. **...* ****

BbVV-1 (HE572591)       ELCKHVGMPDWYRDVLVKSFDRIYYTDNNGRHKIAGTLMSGHRATTFINSVLNAAYIRAAIGSGRFDSLLSL
       EABb 01/103Su    ELCKHVGMPDWYRDVLVKSFDRIYYTDSDGRHKIAGTLMSGHRATTFINSVLNAAYIRAAIGSGRFDSLLSL
       EABb 01/12Su     ELCKHVGMPDWYRDVLVKSFDRIYYTDSDGRHKIAGTLMSGHRATTFINSVLNAAYIRAAIGSGRFDSLLSL
       EABb 00/88Su     ELCKHVGMPDWYRDVLVKSFDRIYYTDSDGRHKIAGTLMSGHRATTFINSVLNAAYIRAAIGSGRFDSLLSL
       EABb 00/23Su     ELCKHVGMPDWYRNVLVKSFDRIYYTDGDGRHKIAGTLMSGHRATTFINSVLNAAYIRAAIGSGRFDSLLSL
BbVV-2 (NC_024151)      VLCERVGAPKWYSRVLRDSFDKEYITIGSDRMHVKGTLMSGHRGTTFINSVLNAAYLRHALGGHYFDSLPSI
                         **::** *.**  ** .***: * * ...* :: ********.************:* *:*.  **** *:
```

S3 Fig. Victoriviruses in *Beauveria bassiana*. (a) 1% (w/v) agarose gel electrophoresis of dsRNA extracted from *B. bassiana* isolate EABb 01/103Su harboring BbVV-1 using virus purification (lane 3). Lane 1 contains the DNA marker Hyperladder I (Bioline), the sizes of which are shown to the left of the gel. **(b)** Schematic representation of the genomic organisation of BbVV-1. The BbVV-1 genome consists of a single dsRNA that contains two overlapping ORFs encoding a CP (light grey box) and an RdRP (dark grey box) flanked by 5'- and 3'-UTRs (black boxes). In the alignment of partial RdRP sequences of members of the genus *Victorivirus*, family *Totiviridae* isolated from *B. bassiana* strains, including BbVV-1 (accession number HE572591; Herrero et al. 2012) and BbVV-2 (accession number NC_024151; Yie et al. 2014), asterisks signify identical aa residues, colons signify highly conserved residues and single dots signify less conserved but related residues.
