# Supplementary material for: Studies on the Virome of the Entomopathogenic Fungus Beauveria bassiana Reveal Novel dsRNA Elements and Mild Hypervirulence
Source: PLoS Pathog. 2017 Jan 23;13(1):e1006183. doi: 10.1371/journal.ppat.1006183 (PMC5293280; doi:10.1371/journal.ppat.1006183)
Supplement: S4 Fig — Schematic representation of the genomic organisation of (a) BdPmV-1 and (b) CcPmV-1. Each genome consist of five dsRNAs, each containing one ORF (grey boxes) flanked by 5’- and 3’-UTRs (black boxes). A comparative alignment of the 5’- and 3’-terminal sequences of (c) BdPmV-1 dsRNAs 1–5, (d) CcPmV-1 dsRNAs 1–5, (e) AltPmV-1 dsRNAs 2, 5 and 6, (f) BbPmV-1 dsRNAs 1–4 and (g) BbPmV-1 dsRNAs 1, 6 and 7. Asterisks signify identical nucleotides. AltPmV-1 dsRNA 6 does not share a similar 3’-terminal sequence with dsRNAs 2 or 5; however there is an internal sequence in its 3’-UTR with significant similarity to the 3’-terminus of dsRNAs 2 and 5, indicating a possible sequencing error. Virus names, acronyms, and GenBank accession numbers are listed in S3 Table. (PDF) [file ppat.1006183.s007.pdf]

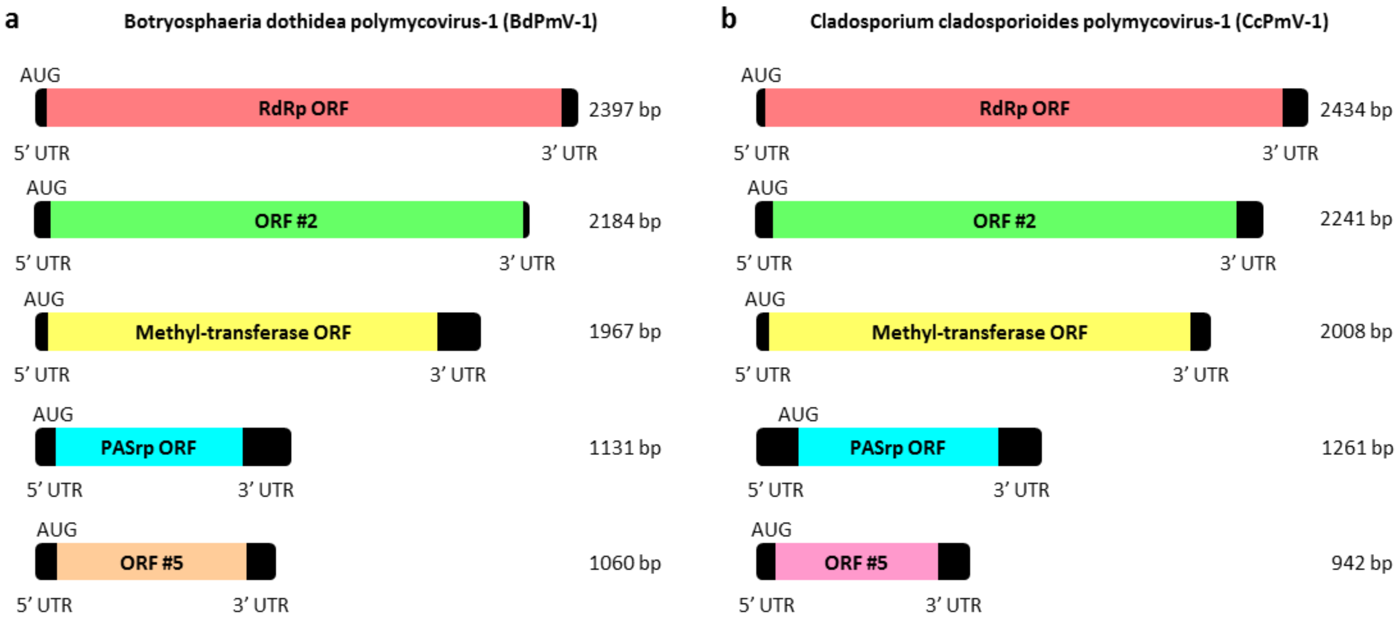

c

```
BdPmV-1 5' UTR termini
dsRNA 1 (1-53 bp)      CGATTAAAACTTTAGTTATTCGCTTGTTCCCTACGTCGCGAGGTTATCTAAGC-----------------------
dsRNA 2 (1-66 bp)      CGATTAAAACTTTAGTTTTTCGCTTGTTCCGGACGTCGCGAGGTTCCTTGAAGGCGTAGAACGTTC----------
dsRNA 3 (1-57 bp)      CGATTAAAACTTTAGTTTTTAGCTTGCCCCCAACGTCGCAAAGGCCCTCGGTTACAA-------------------
dsRNA 4 (1-77 bp)      CGATTAAAATTTTAAATATCGGCTTGTTCCCAACGTCGCAGCAGAGCCACCAGGCAAATTGCAAAAACGACTCGGAA
dsRNA 5 (1-77 bp)      CGATTAAAATTTAACATATCGGCTTGTTCCGTCTGTCGTAAGGTCCTTTCCAGGCAACTCACGTTCACCTACGGAAA
                       *********.** *  * *. *****..**   .****... .   ..

BdPmV-1 3' UTR termini
dsRNA 1 (2419-2434 bp)  CCCCCCGCCCCGGATGGGGT
dsRNA 2 (2226-2241 bp)  CCCTCGCCGTGGGGTGGGGT
dsRNA 3 (1996-2008 bp)  CCCCCGGCGGCGGGTGGGGT
dsRNA 4 (1251-1261 bp)  CCTCGCGGGGGGGATGGGGT
dsRNA 5 (930-942 bp)    CCCCCGCCGGCGCATGGGGT
                       **..       * .******
```

d

```
CcPmV-1 5' UTR termini
dsRNA 1 (1-43 bp)      CGTAAATAAACCTTAACCGCTACAAGTATTATCAGATACGAGC----------------------------------
dsRNA 2 (1-68 bp)      CGTAAATAAACCTTAACCTGTACAAGTATAATCAGTATTGAGGTTCCCAACCTCATTGCCTCGCGAAG---------
dsRNA 3 (1-57 bp)      CGTAAATAAACCTTAACCACTACAAGTATTATCAGATACGAGAAACGCACCTCGACC--------------------
dsRNA 4 (1-77 bp)      CGTAAATAAACCTTAACCGCTACAAGCATTCCCAGAGTCCAGAAAGTGACTCTGCGTAACCTGCGAAACTCCGTTTC
dsRNA 5 (1-76 bp)      -GTAAATAAACCTTAACCGCAGCAAGTATACCACAAGGTGTAAAAGACACTCCCGTTCTTTTACCCCTTGTCGTTTC
                       *****************    .****.**  .  .   .  .

CcPmV-1 3' UTR termini
dsRNA 1 (2421-2434 bp)  TGGGGGGGGGGGG-
dsRNA 2 (2228-2241 bp)  TGGGGGAGGGGGG-
dsRNA 3 (1995-2008 bp)  CCGTGGGGGGGGG-
dsRNA 4 (1247-1261 bp)  CCGCCGCGGGGGGGA
dsRNA 5 (929-942 bp)    CCGTGACGGGGGGG-
                       . *  . *******
```

e

```
AltPmV-1 5' UTR termini
dsRNA 2 (1-71 bp)      CGAACAAACCAGTTCTTCTGTACTTCGTAGCTACTTCGTCTGGTTTCCGGTCCAACAAAACCCCTTGAGTG-
dsRNA 5 (1-72 bp)      TGAACAAACCAGTTCTTCTGCATTTGTAGGCTTTATCGTCCTGTTTCCGTAGGCACAGACCTTGTAACACGA
dsRNA 6 (1-72 bp)      TGAACAAACCATAACTTCCGTACAAGTACACTACGTCGTTTGTTCCCCGGAGAAACAAGAGTCAAGAAAGGA
                       .**********   ****.*.*.      .** . ****..  *..***     ***..  ..   . . *

AltPmV-1 3' UTR termini
dsRNA 2 (2206-2233 bp)  GTTCCGGGCCCCAGACGGGGTGGAGTTT
dsRNA 5 (1338-1365 bp)  GCCCGCTCCCCCAGACGGGGTGGAATTT
dsRNA 6 (860-887 bp)    CTTCCTTGTTTCGGCGAGGGGGGGGTTT
                        ..*    ...*.*  .*** **..***
```

f

```
BbPmV-1 5' UTR termini
dsRNA 1 (1-26 bp)      CGAATATATATTTTAATTTCGG-CAA-C---------------------------------------------
dsRNA 2 (1-71 bp)      CGAATATATATTTTAATTTT-GTCAAGCCATCAGTACTGTGGGCCACTCCCATAGGCCGTCTTGAGATTGGA
dsRNA 3 (1-31 bp)      CGAATATATATTTTAATTTTCG-CAAGCCATC-----------------------------------------
dsRNA 4 (1-72 bp)      CGAATATATATTCAAAACTTTGTCTTGCCATCGGTAAAGGGAGCTACACCAATAGCCCCAACCGTGTTCGAG
                       ************. ** .*. * *   *

BbPmV-1 3' UTR termini
dsRNA 1 (2414-2425 bp)  CATAGTTTTTTT-
dsRNA 2 (2251-2260 bp)  CGTAGTTTTT---
dsRNA 3 (1909-1921 bp)  CATAGTTTTTTTG
dsRNA 4 (1364-1373 bp)  CGTAGTTTTG---
                       *.*******
```

g

```
BbPmV-2 5' UTR termini
dsRNA 1 (1-26 bp)      CGAACTAAAGAGTTTTTCTACACAAG
dsRNA 6 (1-26 bp)      CGAACTAAAGGCTTTCTTTTCACAAG
dsRNA 7 (1-26 bp)      CGAACAAAAGAGTTTTTCTACGCAAG
                       ***** ****. ***.*.* *.****

BbPmV-2 3' UTR termini
dsRNA 1 (2397-2405 bp)  GGAATTTTT
dsRNA 6 (1001-1009 bp)  GTGAATTTT
dsRNA 7 (678-686 bp)    GAATTTTTT
                       * .  ****
```

S4 Fig. Sequence properties of known polymycoviruses. Schematic representation of the genomic organisation of **(a)** BdPmV-1 and **(b)** CcPmV-1. Each genome consist of five dsRNAs, each containing one ORF (grey boxes) flanked by 5'- and 3'-UTRs (black boxes). A comparative alignment of the 5'- and 3'-terminal sequences of **(c)** BdPmV-1 dsRNAs 1–5, **(d)** CcPmV-1 dsRNAs 1–5, **(e)** AltPmV-1 dsRNAs 2, 5 and 6, **(f)** BbPmV-1 dsRNAs 1–4 and **(g)** BbPmV-1 dsRNAs 1, 6 and 7. Asterisks signify identical nucleotides. AltPmV-1 dsRNA 6 does not share a similar 3'-terminal sequence with dsRNAs 2 or 5; however there is an internal sequence in its 3'-UTR with significant similarity to the 3'-terminus of dsRNAs 2 and 5, indicating a possible sequencing error. Virus names, acronyms, and GenBank accession numbers are listed in S3 Table.
