# Supplementary material for: Studies on the Virome of the Entomopathogenic Fungus Beauveria bassiana Reveal Novel dsRNA Elements and Mild Hypervirulence
Source: PLoS Pathog. 2017 Jan 23;13(1):e1006183. doi: 10.1371/journal.ppat.1006183 (PMC5293280; doi:10.1371/journal.ppat.1006183)
Supplement: S5 Fig — (a) Comparison of the conserved motifs of the RdRP in polymycoviruses. Numbers within the brackets indicate the number of aa not shown. In the RdRP motifs, the symbol ‘#’ signifies S or T and the symbol ‘&’ signifies bulky hydrophobic residues (I, L, V, M, F, Y, W). In all sequence alignments, asterisks signify identical aa residues, colons signify highly conserved residues and single dots signify less conserved but related residues. (b) Comparison of the cysteine-rich zinc finger-like found in the proteins encoded by dsRNA 2 of polymycoviruses. (c) Comparison of the catalytic methyltransferase motifs found in the proteins encoded by dsRNA 3 of polymycoviruses and 5’-RLM RACE oligo-cap analysis of BbPmV-1 dsRNA 1. Viral dsRNA was (i) treated with CIP and TAP and ligated to an oligoribonucleotide adaptor at the 5’-terminus, (ii) treated with CIP only and ligated, (iii) left untreated and ligated and (iv) left untreated and unligated. Subsequently, RT-PCR was performed with a primer homologous to the oligonucleotide adaptor and a sequence-specific primer, and the products were electrophoresed on a 4.5% (w/v) native polyacrylamide gel. The expected product size is 310 bp, indicated by an arrow, and the positive reaction in lane 1 indicates that the positive-strand of dsRNA 1 is capped. (d) Proline-alanine-serine (PAS) content of the proteins encoded by RNA 4 of polymycoviruses. (e) PAS content of the proteins encoded by ORF-1 of unirnaviruses. (f) AFM image of the non-conventionally encapsidated viruses from the EABb 92/11-Dm isolate. BbPmV-1 and BbNV-1 are visualized as chain-like linear nucleic acids of different lengths corresponding to those predicted from the size of the genomic dsRNAs. (g) PAS content of the proteins encoded by the extra dsRNAs of polymycoviruses. Virus names, acronyms, and GenBank accession numbers are listed in S3 Table. (PDF) [file ppat.1006183.s008.pdf]

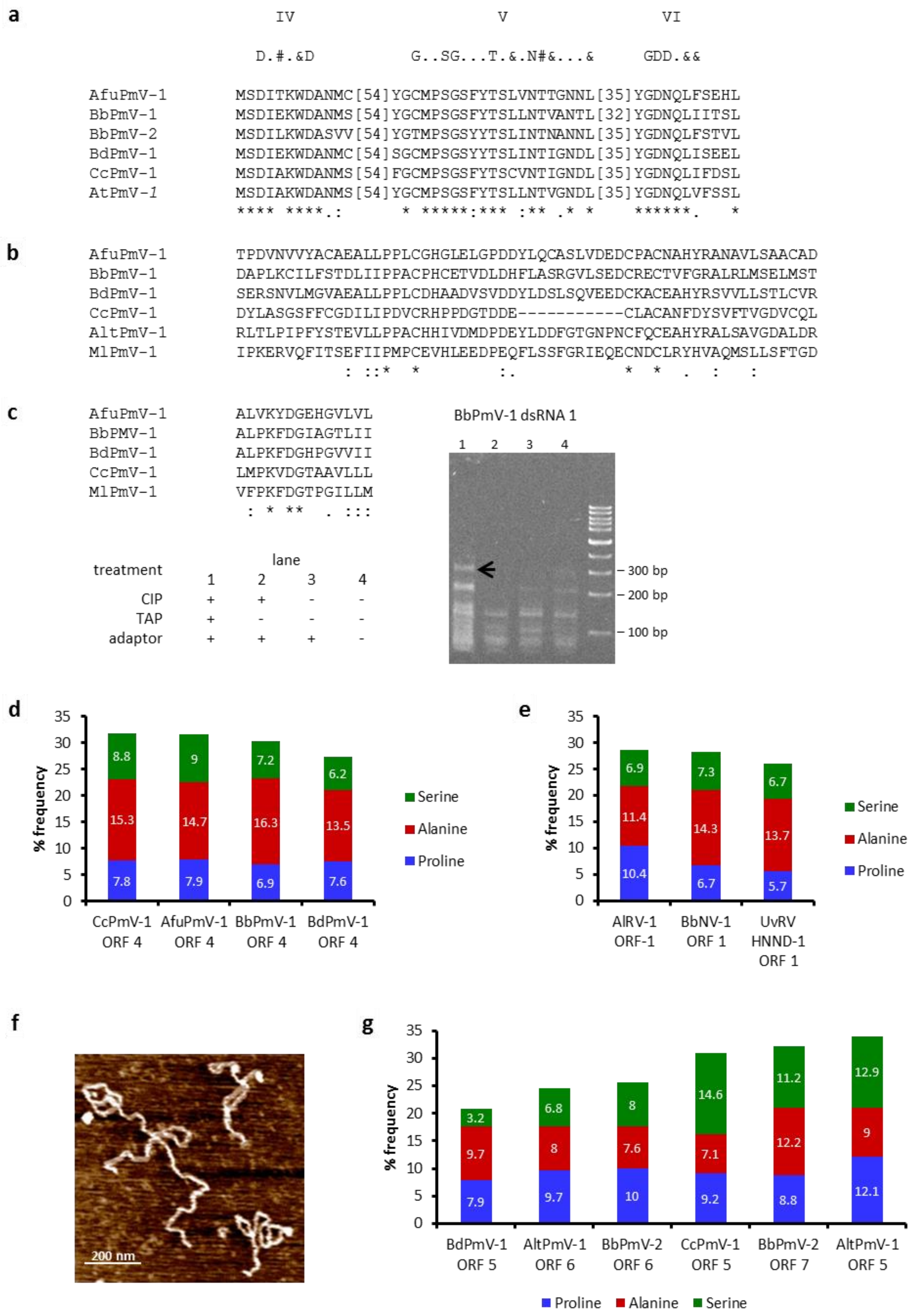

S5 Fig. Sequence and functional analysis of the polymycovirus-encoded proteins. (a) Comparison of the conserved motifs of the RdRP in polymycoviruses. Numbers within the brackets indicate the number of aa not shown. In the RdRP motifs, the symbol '#' signifies S or T and the symbol '&' signifies bulky hydrophobic residues (I, L, V, M, F, Y, W). In all sequence alignments, asterisks signify identical aa residues, colons signify highly conserved residues and single dots signify less conserved but related residues. **(b)**

Comparison of the cysteine-rich zinc finger-like found in the proteins encoded by dsRNA 2 of polymycoviruses. **(c)** Comparison of the catalytic methyltransferase motifs found in the proteins encoded by dsRNA 3 of polymycoviruses and 5'-RLM RACE oligo-cap analysis of BbPmV-1 dsRNA 1. Viral dsRNA was (i) treated with CIP and TAP and ligated to an oligoribonucleotide adaptor at the 5'-terminus, (ii) treated with CIP only and ligated, (iii) left untreated and ligated and (iv) left untreated and unligated. Subsequently, RT-PCR was performed with a primer homologous to the oligonucleotide adaptor and a sequence-specific primer, and the products were electrophoresed on a 4.5% ($^{w}/_{v}$) native polyacrylamide gel. The expected product size is 310 bp, indicated by an arrow, and the positive reaction in lane 1 indicates that the positive-strand of dsRNA 1 is capped. **(d)** Proline-alanine-serine (PAS) content of the proteins encoded by RNA 4 of polymycoviruses. **(e)** PAS content of the proteins encoded by ORF-1 of unirnaviruses. **(f)** AFM image of the non-conventionally encapsidated viruses from the EABb 92/11-Dm isolate. BbPmV-1 and BbNV-1 are visualized as chain-like linear nucleic acids of different lengths corresponding to those predicted from the size of the genomic dsRNAs. **(g)** PAS content of the proteins encoded by the extra dsRNAs of polymycoviruses. Virus names, acronyms, and GenBank accession numbers are listed in S3 Table.

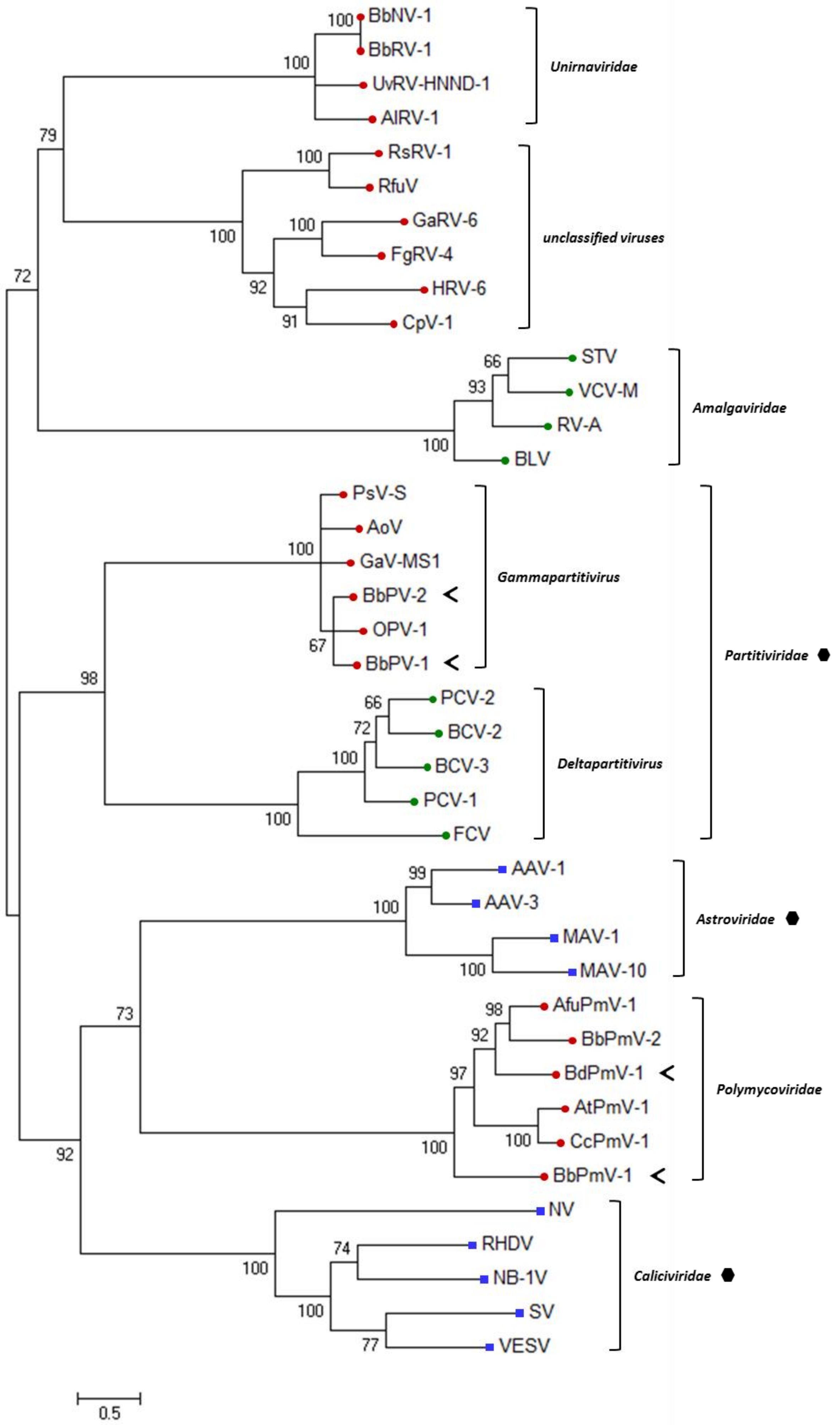
BbNV-1
BbRV-1
UvRV-HNND-1
AlRV-1
RsRV-1
RfuV
GaRV-6
FgRV-4
HRV-6
CpV-1
STV
VCV-M
RV-A
BLV
PsV-S
AoV
GaV-MS1
BbPV-2
OPV-1
BbPV-1
PCV-2
BCV-2
BCV-3
PCV-1
FCV
AAV-1
AAV-3
MAV-1
MAV-10
AfuPmV-1
BbPmV-2
BdPmV-1
AtPmV-1
CcPmV-1
BbPmV-1
NV
RHDV
NB-1V
SV
VESV
Unirnaviridae
unclassified viruses
Amalgaviridae
Gammapartitivirus
Deltapartitivirus
Partitiviridae
Astroviridae
Polymycoviridae
Caliciviridae
0.5

S6 Fig. Maximum likelihood phylogenetic tree created based on the alignment of RdRP sequences of polymycoviruses and related viruses belonging to the Superfamily 1 (Tables S2-3) using the rtREV+G+I+F substitution model. Branches with bootstrap support lower that 50% were collapsed. At the end of the branches, circles indicate that the virus has a dsRNA genome and squares indicate that the virus has a ssRNA genome. Red, green and blue indicate that the virus infects fungi, plants and vertebrates respectively. Next to the virus family name the presence of a hexagon indicates that members of the family are known to be conventionally encapsidated. BbPmV-1 and BbPmV-2 are indicated by arrows.

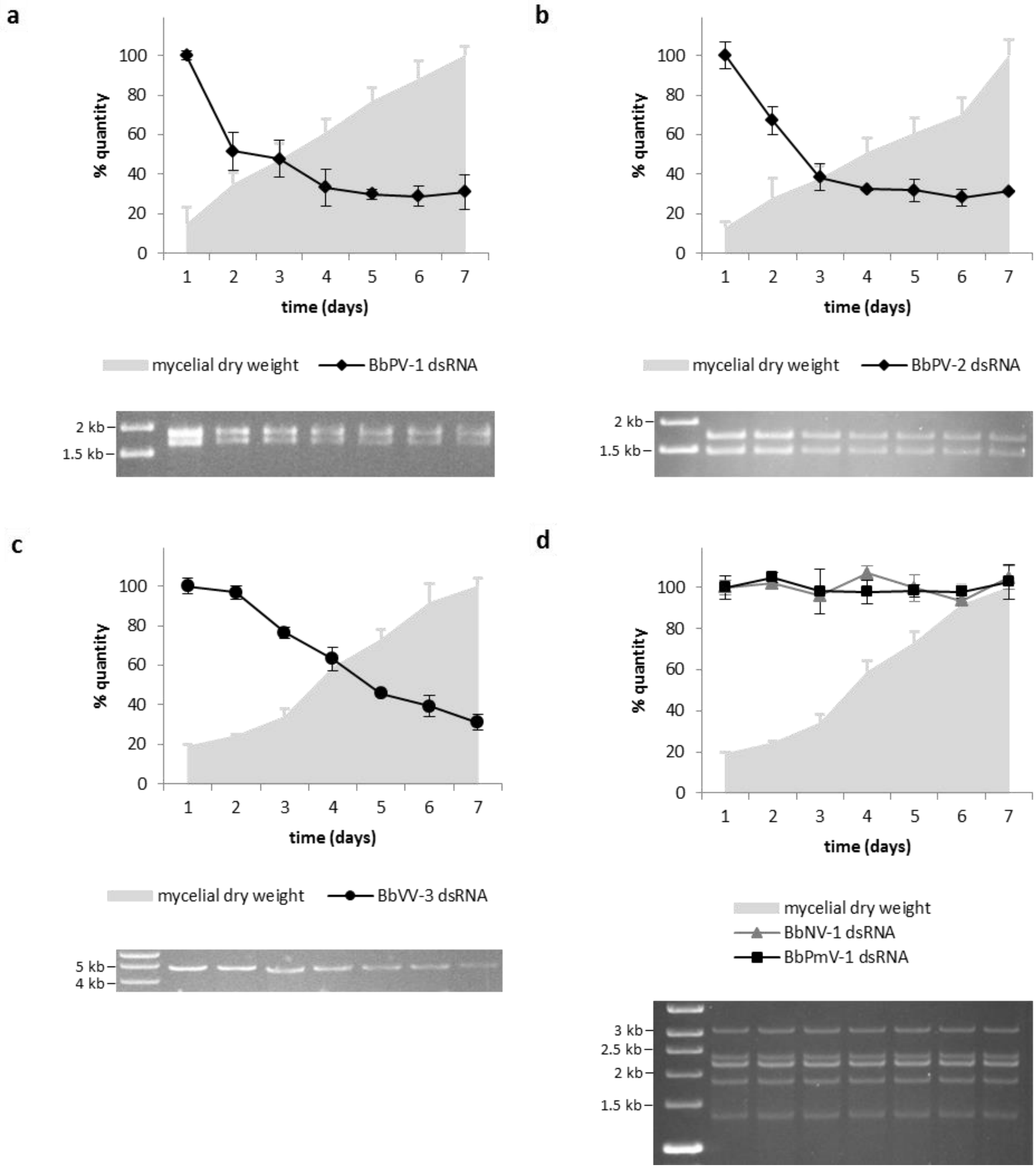

S7 Fig. Time course study of viral dsRNA levels correlated with fungal growth. Biomass production of isolates **(a)** IMI 331273, **(b)** IMI 392612, **(c)** EABb 01/103Su and **(d)** EABb 92/11-Dm in liquid Czapek-Dox CM was assessed daily for 7 days (grey shading). **(a)** BbPV-1, **(b)** BbPV-2, **(c)** BbVV-3, **(d)** BbNV-1 and BbPmV-1 dsRNAs extracted from equal amounts of dry mycelia were electrophoresed in 1% ($^{w}/_{v}$) agarose gels, dsRNA levels were quantified by ImageJ and the results are presented in graphical form (black lines). At least three independent repetitions were performed in duplicate and error bars represent standard deviation. Representative agarose gel analyses of the dsRNAs are shown below each graph.
