# Supplementary material for: Studies on the Virome of the Entomopathogenic Fungus Beauveria bassiana Reveal Novel dsRNA Elements and Mild Hypervirulence
Source: PLoS Pathog. 2017 Jan 23;13(1):e1006183. doi: 10.1371/journal.ppat.1006183 (PMC5293280; doi:10.1371/journal.ppat.1006183)
Supplement: S6 Fig — Maximum likelihood phylogenetic tree created based on the alignment of RdRP sequences of polymycoviruses and related viruses belonging to the Superfamily 1 (S2 and S3 Tables) using the rtREV+G+I+F substitution model. Branches with bootstrap support lower that 50% were collapsed. At the end of the branches, circles indicate that the virus has a dsRNA genome and squares indicate that the virus has a ssRNA genome. Red, green and blue indicate that the virus infects fungi, plants and vertebrates respectively. Next to the virus family name the presence of a hexagon indicates that members of the family are known to be conventionally encapsidated. BbPmV-1 and BbPmV-2 are indicated by arrows. (PDF) [file ppat.1006183.s009.pdf]

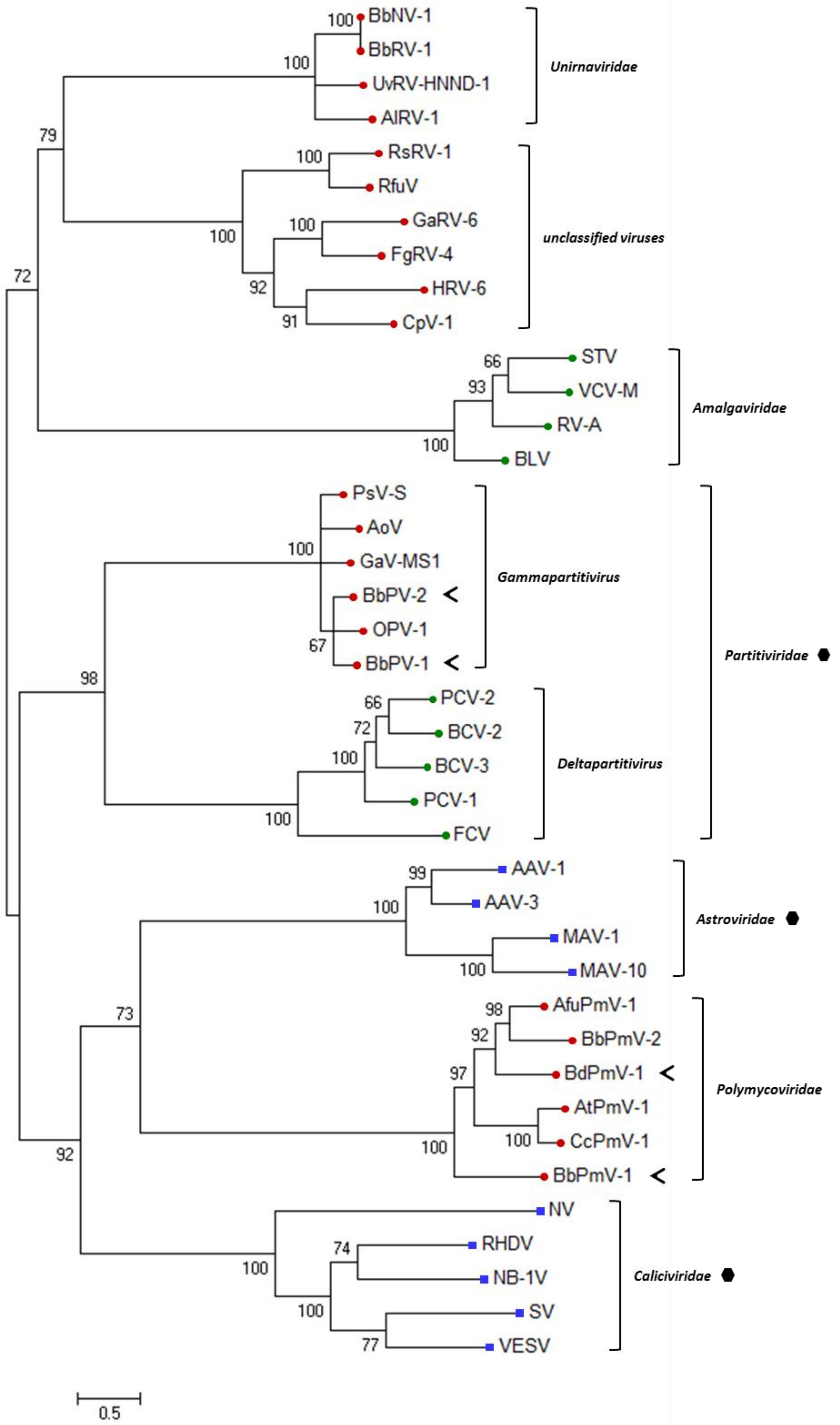

BbNV-1
BbRV-1
UvRV-HNND-1
AlRV-1
RsRV-1
RfuV
GaRV-6
FgRV-4
HRV-6
CpV-1
STV
VCV-M
RV-A
BLV
PsV-S
AoV
GaV-MS1
BbPV-2
OPV-1
BbPV-1
PCV-2
BCV-2
BCV-3
PCV-1
FCV
AAV-1
AAV-3
MAV-1
MAV-10
AfuPmV-1
BbPmV-2
BdPmV-1
AtPmV-1
CcPmV-1
BbPmV-1
NV
RHDV
NB-1V
SV
VESV
Unirnaviridae
unclassified viruses
Amalgaviridae
Gammapartitivirus
Deltapartitivirus
Partitiviridae
Astroviridae
Polymycoviridae
Caliciviridae
0.5

S6 Fig. Phylogenetic analysis of polymycoviruses. Maximum likelihood phylogenetic tree created based on the alignment of RdRP sequences of polymycoviruses and related viruses belonging to the Superfamily 1 (S2-3 Tables) using the rtREV+G+I+F substitution model. Branches with bootstrap support lower that 50% were collapsed. At the end of the branches, circles indicate that the virus has a dsRNA genome and squares indicate that the virus has a ssRNA genome. Red, green and blue indicate that the virus infects fungi, plants and vertebrates respectively. Next to the virus family name the presence of a hexagon indicates that members of the family are known to be conventionally encapsidated. BbPmV-1 and BbPmV-2 are indicated by arrows.
