# Supplementary material for: Studies on the Virome of the Entomopathogenic Fungus Beauveria bassiana Reveal Novel dsRNA Elements and Mild Hypervirulence
Source: PLoS Pathog. 2017 Jan 23;13(1):e1006183. doi: 10.1371/journal.ppat.1006183 (PMC5293280; doi:10.1371/journal.ppat.1006183)
Supplement: S7 Fig — (a) 1% (w/v) agarose gel electrophoresis and northern blot hybridization of dsRNA extracted from the EABb 92/11-Dm and EABb 92/11-DmC1 isogenic lines. Hybridization was carried out using a probe specific for BbPmV-1 dsRNA1. Attempted RT-PCR amplification of (b) a 838 bp BbPmV-1 dsRNA1 fragment and (c) a 502 bp BbNV-1 dsRNA fragment from the EABb 92/11-Dm, EABb 92/11-DmC1, EABb 92/11-DmC2, EABb 92/11-DmC3 and EABb 92/11-DmT isogenic lines. In all gels, lane 1 contains the DNA marker Hyperladder I (Bioline), the sizes of which are shown to the left of the gel. Comparison of the fungal growth in solid and liquid medium; (d) radial growth and (e) biomass production of EABb 92/11-Dm, EABb 92/11-DmC1, EABb 92/11-DmC2 and EABb 92/11-DmC3. At least three independent repetitions were performed in triplicate (radial growth) or duplicate (biomass production) and error bars represent standard deviation. A yellow background indicates the time points when the difference in growth is statistically significant (Student’s t test, P-value < 0.05). (f) Mean survival curves of G. mellonella larvae infected with the EABb 92/11-Dm, EABb 92/11-DmC1, EABb 92/11-DmC2 and EABb 92/11-DmC3 isogenic lines. At least three independent repetitions were performed in triplicate. (PDF) [file ppat.1006183.s010.pdf]

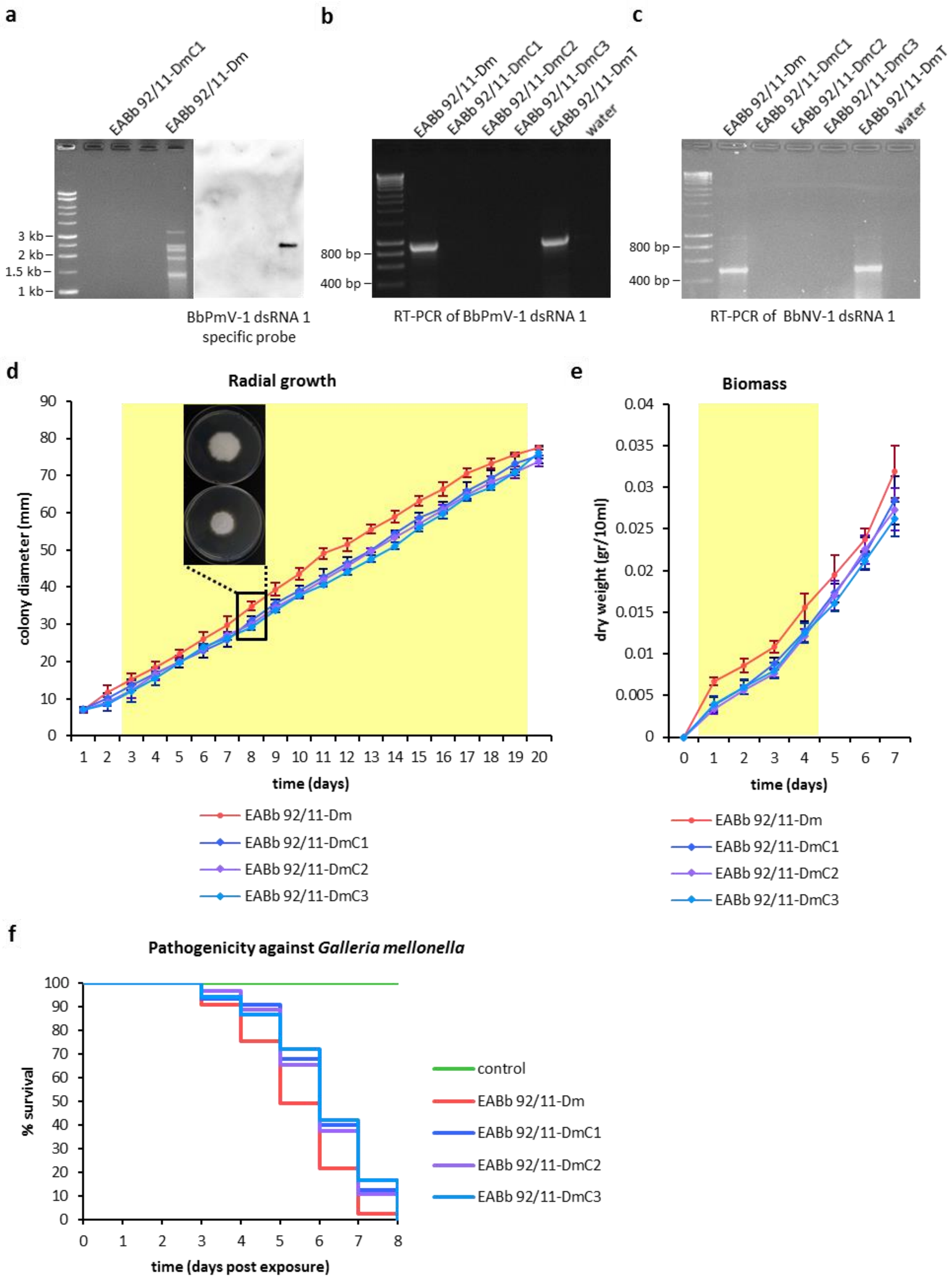

S7 Fig. Comparison of the *Beauveria bassiana* EABb 92/11-Dm and EABb 92/11-DmC; virus-infected and virus-free isogenic lines. **(a)** 1% ($^{w}/_{v}$) agarose gel electrophoresis and northern blot hybridization of dsRNA extracted from the EABb 92/11-Dm and EABb 92/11-DmC1 isogenic lines. Hybridization was carried out using a probe specific for BbPmV-1 dsRNA1. Attempted RT-PCR amplification of **(b)** a 838 bp BbPmV-1 dsRNA1 fragment and **(c)** a 502 bp BbNV-1 dsRNA fragment from the EABb 92/11-Dm, EABb 92/11-DmC1, EABb 92/11-DmC2, EABb 92/11-DmC3 and EABb 92/11-DmT isogenic lines. In all gels, lane 1 contains the DNA

marker Hyperladder I (Bioline), the sizes of which are shown to the left of the gel. Comparison of the fungal growth in solid and liquid medium; **(d)** radial growth and **(e)** biomass production of EABb 92/11-Dm, EABb 92/11-DmC1, EABb 92/11-DmC2 and EABb 92/11-DmC3. At least three independent repetitions were performed in triplicate (radial growth) or duplicate (biomass production) and error bars represent standard deviation. A yellow background indicates the time points when the difference in growth is statistically significant (Student's t test, P-value < 0.05). **(f)** Mean survival curves of *G. mellonella* larvae infected with the EABb 92/11-Dm, EABb 92/11-DmC1, EABb 92/11-DmC2 and EABb 92/11-DmC3 isogenic lines. At least three independent repetitions were performed in triplicate.
