# Supplementary material for: Studies on the Virome of the Entomopathogenic Fungus Beauveria bassiana Reveal Novel dsRNA Elements and Mild Hypervirulence
Source: PLoS Pathog. 2017 Jan 23;13(1):e1006183. doi: 10.1371/journal.ppat.1006183 (PMC5293280; doi:10.1371/journal.ppat.1006183)
Supplement: S8 Fig — Biomass production of isolates (a) IMI 331273, (b) IMI 392612, (c) EABb 01/103Su and (d) EABb 92/11-Dm in liquid Czapek-Dox CM was assessed daily for 7 days (grey shading). (a) BbPV-1, (b) BbPV-2, (c) BbVV-3, (d) BbNV-1 and BbPmV-1 dsRNAs extracted from equal amounts of dry mycelia were electrophoresed in 1% (w/v) agarose gels, dsRNA levels were quantified by ImageJ and the results are presented in graphical form (black lines). At least three independent repetitions were performed in duplicate and error bars represent standard deviation. Representative agarose gel analyses of the dsRNAs are shown below each graph. (PDF) [file ppat.1006183.s011.pdf]

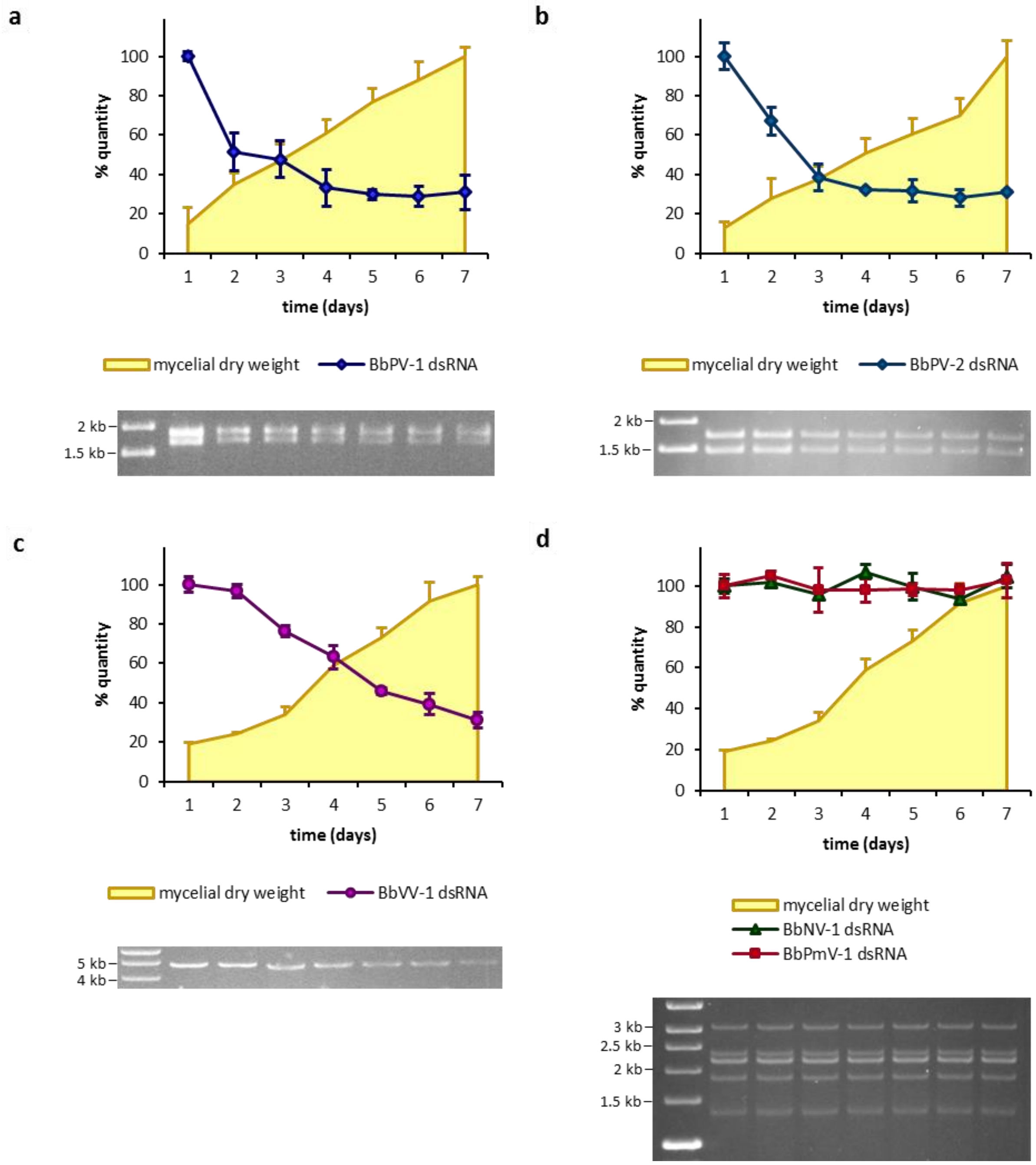

S8 Fig. Time course study of viral dsRNA levels correlated with fungal growth. Biomass production of isolates **(a)** IMI 331273, **(b)** IMI 392612, **(c)** EABb 01/103Su and **(d)** EABb 92/11-Dm in liquid Czapek-Dox CM was assessed daily for 7 days (grey shading). **(a)** BbPV-1, **(b)** BbPV-2, **(c)** BbVV-3, **(d)** BbNV-1 and BbPmV-1 dsRNAs extracted from equal amounts of dry mycelia were electrophoresed in 1% ($^{w}/_{v}$) agarose gels, dsRNA levels were quantified by ImageJ and the results are presented in graphical form (black lines). At least three independent repetitions were performed in duplicate and error bars represent standard deviation. Representative agarose gel analyses of the dsRNAs are shown below each graph.
